# Hypertension and epigenetic aging: socio-biological pathways of health disparity

**DOI:** 10.1186/s13148-026-02117-w

**Published:** 2026-04-04

**Authors:** Su Yon Jung

**Affiliations:** 1 Translational Sciences Section, School of Nursing, University of California, 700 Tiverton Ave. 3-264 Factor Building, Los Angeles, CA 90095 USA; 2 Department of Epidemiology, Fielding School of Public Health, University of California, Los Angeles, Los Angeles, CA 90095 USA; 3 Jonsson Comprehensive Cancer Center, University of California, Los Angeles, Los Angeles, CA 90095 USA

**Keywords:** Hypertension, Epigenetic aging clock, Aging acceleration, DNA methylation, Social determinants of health, Health disparity, African American, Postmenopausal women

## Abstract

**Background:**

Hypertension incidence increases with age, with its highest burden in elderly African Americans (AAs). But socio-biological aging pathways underlying this difference are not well characterized, with a substantial paucity of epigenetic studies on hypertension-related racial disparity in the elderly. We primarily examined epigenetic aging acceleration (aging accel) in the prospective development of hypertension and secondarily investigated the extent to which adverse social exposures mediated AAs’ greater aging accel and, jointly with increased aging accel, explained their greater risk of hypertension outcomes.

**Methods:**

We obtained global-level DNA methylation and clinical data, social determinants of health (SDOH), and physiological data from > 1,500 postmenopausal women in the Women’s Health Initiative. Hypertension outcomes were followed for 3-year short-term and a mean of 17-year longer-term periods. Levine’s aging accel was estimated, and mediation analyses were performed with SDOH and aging accel via Sobel and Multiple Mediation Analysis.

**Results:**

Greater epigenetic aging was associated with hypertension outcomes during the short-term follow-up. While the lower level of SDOH was related to greater aging accel, its influence on greater aging accel in AAs than whites varied by SDOH type and, in combination, was partial (range, 1–26%). Finally, a group of SDOH and aging accel, both separately and jointly, mediated AAs’ greater incidence of hypertension to a limited extent during both short-term and longer-term follow-up periods.

**Conclusions:**

Our study contributes to better understanding of socio-biological hypertensive pathways shared by aging processes, which may inform risk stratification in the elderly in hypertension prevention and racial disparity.

**Supplementary Information:**

The online version contains supplementary material available at 10.1186/s13148-026-02117-w.

## Background

Hypertension is key to cardiovascular diseases (CVDs) such as coronary heart disease, myocardial infarction, and stroke [1, 2], but the risk factors for early prediction of hypertension are not well established. Hypertension incidence increases with age, occurring in approximately 75% of those older than 60 years (data from the 2017–2018 [3]), and African Americans (AAs) have the greatest burden of hypertension among older adults in the U.S [3, 4]. AAs are also disproportionately affected by a greater incidence of aortic stiffness, one central component of vascular aging and an underlying factor for hypertension [5].

Increased blood pressure (BP) variability, exerting its impact on hypertension, shares molecular pathways with those of aging, including genomic instability, epigenetic modification, mitochondrial oxidative damage, and cellular senescence [6]. Inter-individual differences in vascular aging may be a consequence of the different rate of biological aging acceleration (aging accel) between individuals even at the same chronological age. Biological aging can be measured with various biomarkers at both the molecular and cellular levels, such as telomere attrition, protein homeostasis and, more recently, epigenetics; they may accurately assess an individual’s aging-related diseases [7, 8]. DNA methylation (DNAm), the best-known epigenetic alteration, is a durable and reversable novel biomarker of biological aging, outperforming other aging biomarkers in association with aging, longevity, and aging-related diseases [9]. Indeed, DNAm markers reflect both genetic and environmental effects on cellular functions, playing a key role in regulating gene expression, which is known to change with aging [10]. Epigenetic aging accel, measured as the discrepancy between DNAm-based epigenetic age and chronological age, has thus been a strong prediction matrix for aging-attributable physical disorders such as neurocognitive diseases [11], frailty [12], all-cause mortality [13], certain types of cancer [14, 15], and vascular disorders such as CVD [14].

Of note, the aging process differs by race. In particular, among females, AAs have the shortest life expectancy (78 years) among other racial and ethnic groups (whites: 81 years, according to the 2015 mortality lifetable statistics) [16], suggesting earlier exposures to health deterioration and hence, faster aging in this disadvantaged group [17]. However, studies of biological aging processes and relevant markers that compare the races are limited. Better characterizing an aging biomarker for deeper understanding of the underlying biological difference in aging may contribute to advanced risk prediction of the disease, so helping alleviate hypertension-related health disparity.

In addition, a sexual disparity exists for hypertension: among younger people, a greater burden of hypertension in men than women, but that shifts with age across races, so that women have greater prevalence of hypertension in an old-age group [3, 18]. Hypertension is the primary hazard factor for CVD and premature death in women worldwide [19]. Nevertheless, a substantial paucity exists in studies for women-specific hypertensive biological pathways, resulting in a lack of understanding of etiology and risk prediction specific to women in clinical preventive medicine [20].

**Fig. 1:**
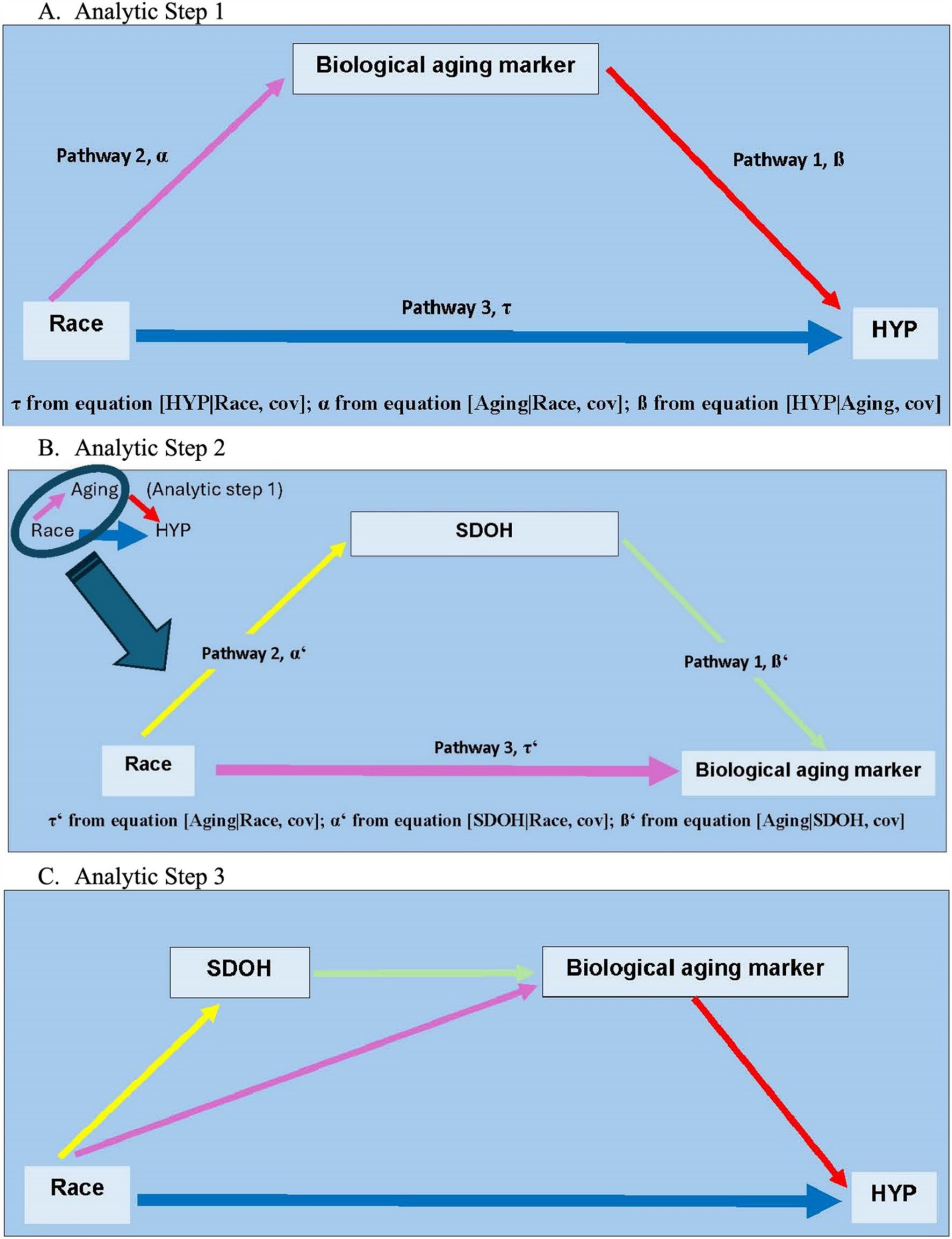
Diagram of modeled analytic steps and relevant pathways. (*HYP* hypertension development., *SDOH* social determinants of health)

In our current study, we addressed these deficits by examining older women who are postmenopausal (ages 50 years and older) for their DNAm-based aging accel in the prospective development of hypertension. We further examined whether the aging accel rate differed between AAs and whites and to what extent the aging disparity mediated the racial difference in hypertension incidence (Fig. 1A). Our primary study objective was to investigate whether greater biological aging was observed in women with hypertension outcomes and in AAs.

Further, cumulative adverse social exposures, reflecting low resources of the social determinants of health (SDOH), aggravate epigenetic aging accel [21, 22] and also promote greater hypertension prevalence [3] and poorer CVD outcomes [23]. On the basis of evidence, our exploratory hypothesis was that a lower level of SDOH contributes to greater aging accel and, via its influence on greater aging accel in AAs than in whites, the SDOH-induced aging accel jointly mediates the greater incidence of hypertension in AAs (Figs. 1B and C). Our study aimed to better understand the biological aging pathway that integrates a critical exposomic factor, SDOH, in the development of hypertension from the viewpoint of racial disease disparity. This may help identify an early-risk group who can benefit from epigenetically informed preventive interventions. Further, elucidating the mechanistic link between an unhealthy environment such as social risk and epigenetic aging can inform preventive public practices to adequately address the unfavorable social exposures for those at epigenetically high risk.

## Materials and methods

### Study population

We included postmenopausal women 50–79 years old at enrollment from the Women’s Health Initiative Database for Genotypes and Phenotypes (WHI-dbGaP) genetic repository. The WHI Study was initiated in 1993, recruiting study participants through 1998 from more than 40 U.S. clinical centers, and has since followed active participants annually to collect clinical and physiological data and health updates [24, 25]. We selected the largest ancillary study, BAA23, which has available peripheral blood lymphocyte (PBL)-based global DNAm data [26]. From a total of 2,107 women, 676 AAs and 998 whites were initially included (Figure S1). With a mean 19-year (12–29 years) long-term follow-up, we excluded non-active participants (*n* = 748) whose data on hypertension status were missing, analyzing a total of 926 (297 AAs and 629 whites) who did not have hypertension at baseline. Among them, 224 (75%) AAs and 425 (67%) whites developed hypertension. In a separate analysis for women who had been followed for only a relatively short period—until their third annual visit (AV3)—we included a total of 1,505 (583 AAs and 922 whites) whose data on hypertension outcomes were available. Among them, 1,039 women did not have hypertension at baseline; of those, 69 of 386 (18%) AAs and 115 of 653 (17%) whites had developed hypertension by AV3. In addition, among 466 women who had had hypertension at baseline, 216 (46%) still had high systolic (> 140 mmHg) and diastolic (≥ 90 mmHg) pressures at AV3. Each WHI clinical center’s institutional review board and the University of California, Los Angeles approved this study.

### Hypertension outcomes and data collection

Clinical, physiological, and self-reported data were collected (Table S1, descriptive summary of following variables): sociodemographic data (age; race; ethnicity; education; family income; any medical insurance; social support), comorbid conditions (diabetes with pill or shots; high cholesterol requiring pills), diets (Healthy Eating Index-2015 [HEI-2015] total score and the scores for fatty acids, whole fruits, and total vegetables components; dietary alcohol intake; frequency of alcohol consumption), lifestyles (physical activity; years of regular smoking), and reproductive history (oophorectomy history; hormone replacement therapy; ages at menopause and menarche). We categorized the following selected SDOH variables (Table S2): education, < college vs. ≥ college; family income, < $20,000, $20,000–$49,999, $50,000–$99,999, and ≥ $100,000; any medical insurance, no vs. yes. Social support was measured as a composite score from 9 questions adapted from the Medical Outcomes Study [27], ranging from 9 to 45, where a greater score indicates more social support. The social support scores were further binary categorized by a median. Anthropometric measurements (height, weight, and waist and hip circumferences) and systolic and diastolic BPs at two different time points per day were measured by trained staff at baseline.

Hypertension development was assessed by self-report of taking pills for hypertension (no vs. yes) during a mean follow-up of 19 years. For the 3-year, relatively short-term follow-up after enrollment, hypertension status and development were determined by using systolic and diastolic BPs: the average of two measurements of systolic BP > 140 mmHg and/or diastolic BP ≥ 90 mmHg was defined as hypertension [28, 29]. Using this BP data obtained at baseline and AV3, we classified the study population into three groups (Table S3): (1) HT.ch.n group, defined as hypertension development status in those without baseline hypertension, categorized as no (reference: no development) vs. yes (development); (2) HT.ch.y group, defined as hypertension improvement status in those with baseline hypertension, categorized as yes (reference: normal BP at AV3) vs. no (continued high BP at AV3); and (3) HT.ch.all group, defined as overall hypertension status change by combining the aforementioned four outcomes, categorized as no development or improvement (reference) vs. development or no improvement.

### Epigenetic clocks and acceleration matrices

Genome-wide DNAm array was conducted on the study population using their PBL-DNA with Illumina 450 BeadChip and was further normalized via beta-mixture quantile [30] as well as batched-adjusted with random intercept for plate and chip and a fixed effect for row [31], yielding a total of 482,421 CpG dinucleotides (CpGs). To address DNAm stability from stored samples [32], we adopted Horvath’s suggestion [33] by controlling leukocyte heterogeneities in estimating DNAm age: CD4^+^ T cells, natural killer cells, monocytes, and granulocytes from Houseman’s method [34] and plasma blasts, CD8^+^CD28^–^CD45RA^–^ T cells, and naïve CD8 T cells from Horvath’s method [33].

PBLs interact with peripheral tissues through excretion of cytokines and other cellular signals, yielding complex regulatory mechanisms inducing hypertension across organ systems [35]. Thus, PBLs are an ideal tissue type for examining epigenetic aging markers and their association with hypertension. Of several epigenetic aging clocks, Levine’s clock outperforms others in risk prediction of mortality and various age-related diseases including CVDs [21, 36, 37] across multiple tissues and organs. Levine’s clock estimates a clinical phenotypic aging score with 513 CpGs based on 9 blood biomarkers, including albumin, creatinine, and C-reactive protein levels [37]. The aging clock is a composite scale from a linear combination of the weighted CpGs at the individual level and was generated by an available online tool [33, 38] and the *methylclock* annotation Bioconductor package.

### Statistical analysis

We estimated epigenetic aging accel, the deviation of epigenetic age from chronological age, by two matrices: (1) AgeAccelDiff, measured by subtracting chronological age from DNAm age and (2) intrinsic epigenetic age acceleration (IEAA), measured by the residual from regressing DNAm age on chronological age, further adjusting for blood cell proportions. The IEAA captures cellular aging accel independently of varied DNAm levels due to cell components’ heterogeneity between individuals [39].

In the analytic step 1 with each aging accel matrix, distributions of the aging accel by hypertension (pathway 1 [path 1, hereafter]) and by race (path 2) were evaluated via unpaired two-sample *t* or Kruskal-Wallis testing if continuous variables were skewed or had outliers. In path 1, the aging accel matrix was regressed on hypertension outcome, reflecting 1-unit increase in aging accel associated with a risk for hypertension incidence; and the aging matrix was further analyzed as a 10-year interval or binary predictor (negative vs. positive aging accel). In the regression analysis of path 2, aging accel matrix was analyzed via continuous or binary outcomes, reflecting the magnitude of the different rate of aging accel by race. In path 3, the result from the logistic regression reflects the degree of racial disparity (whites as reference) in hypertension incidence. For each path’s analysis, we adjusted for the following covariates: (1) Model 1, univariate analysis; (2) Model 2, Model 1 plus body mass index (BMI) and waist-to-hip ratio (WHR); and (3) Model 3, Model 2 plus all other covariates (age, race, diabetes treated with pills or shots, high cholesterol requiring pills, fatty acids, whole fruits, and vegetables, and a total score from HEI-2015, dietary alcohol, alcohol intake frequency, physical activity, years of regular smoking, oophorectomy history, hormone replacement therapy, ages of menopause and menarche, education, family income, any insurance and social support, except tested variables).

In the analytic step 2 with the 3 paths, distribution of individual SDOH by aging accel (path 1) and race (path 2) were examined via unpaired two-sample *t* or Kruskal-Wallis testing as appropriate and, further, race was regressed on each SDOH, indicating the extent of racial difference in the social constructs. In particular, with ordinal income outcomes, we performed the ordinal logistic regression with proportional odds assumption met.

For the mediation effect of SDOH on the greater aging accel in AAs than in whites, a proportional effect of an individual SDOH was estimated via an indirect effect/total effect (= α**‘** x ß**‘** / τ**‘**, refer to Fig. 1B) and tested by the Sobel test. The joint effect by combining SDOH variables was tested via a general Multiple Mediation analytic approach with bootstrapping developed by Yu and Li [40, 41]. Finally, the mediation analyses including total and direct relationships between race and hypertension and an indirect effect on this association via SDOH and aging accel, both separately and jointly, were calculated by the Multiple Mediation Analysis (R packages bda and mma), which was specifically designed to handle scenarios involving multiple mediators [40, 41].

## Results

### Analytic step 1 (Fig. 1A)

**Fig. 2:**
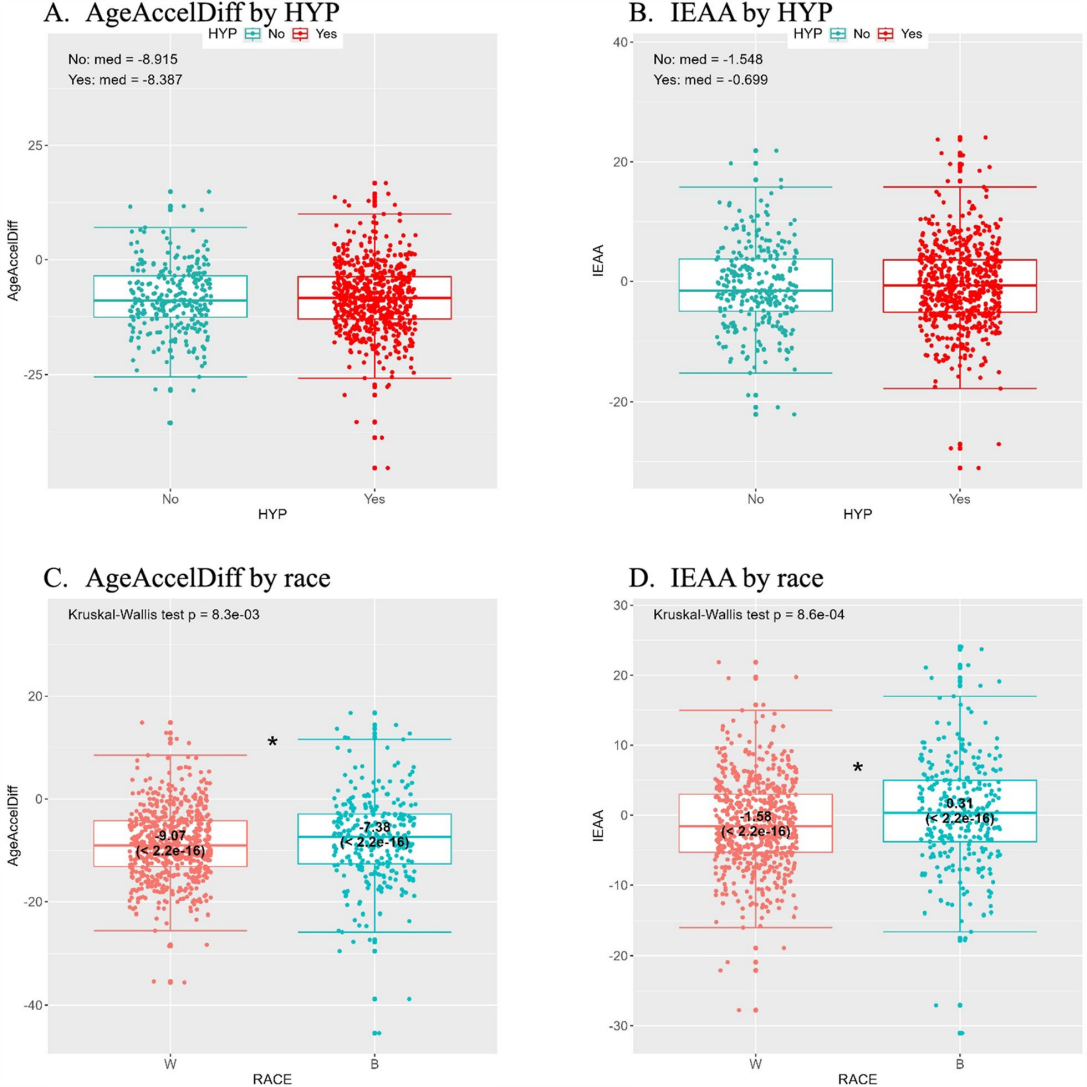
Analytic Step 1: distribution of AgeAccelDiff and IEAA by HYP and race. **A** and **B**, path 1; **C** and **D**, path 2. (AgeAccelDiff, epigenetic age acceleration as departure of DNAmAge from chronological age; B, blacks [African Americans]; HYP, hypertension development; IEAA, intrinsic epigenetic age acceleration as residuals adjusted for cell composition; W, whites)

With the two aging accel matrices (AgeAccelDiff and IEAA) from the hypertension (HYP) group who had been followed for a mean of 19 years, results from the path 1 analysis showed slightly greater aging accel in women who developed hypertension than in women who did not. (Figs. 2A and B; Table 1). Similar results were found in the HT.ch.all group, who had been followed until AV3 for their hypertension status (classified into no hypertension development or improvement vs. development or no improvement) (Figures S2 A and B and Table S4). We further analyzed the DNAm-based age scale itself in this path, yielding a significantly greater DNAm age in the women who developed hypertension or had no improvement at AV3 than in their counterparts (Figure S2 C and Table S4). In the path 2 analysis of both HYP and HT.ch.all groups, AA women had substantially greater aging accel than white women (Figs. 2C and D and Figures S2 D and E), with nearly 60% greater probability of increased aging accel scores (Table 1 and S4). Of note, IEAA scores in both groups remained significantly greater among AAs than whites after the full adjustment of covariates in Model 3. Interestingly, the DNAm age in both the HYP and HT.ch.all groups was lower in AA women than in white women (Figures S2 F and S3 B); but in the HT.ch.all group, after the full adjustment of covariates in Model 3, this association was reversed, showing greater DNAm age in AAs than whites (Tables S4 and S5). Further, in the path 3 analysis of racial difference in hypertension outcomes, greater incidence was observed in AAs than whites in both the HYP and HT.ch.all groups, but statistical significance did not remain in the full models.

**Table 1:** Analytic Step 1 with 3 paths: association of biological aging parameters (AgeAccelDiff and IEAA) with HYP (path 1) and racial difference in biological aging parameter (path 2) and HYP (path 3)

	Model 1*			Model 2**			Model 3***		
< Path 1>	OR	95% CI	*P*	OR	95% CI	*P*	OR	95% CI	*P*
AgeAccelDiff¥	1.003	(0.984, 1.023)	0.761	0.998	(0.979, 1.018)	0.866	1.006	(0.982, 1.031)	0.627
IEAA¥	1.004	(0.983, 1.025)	0.718	0.998	(0.977, 1.019)	0.855	1.003	(0.976, 1.030)	0.835
< Path 2>	Beta	95% CI	*P*	Beta	95% CI	*P*	Beta	95% CI	*P*
AgeAccelDiff€	**1.168**	**(0.161**,** 2.174)**	**0.023**	0.743	(-0.286, 1.773)	0.157	0.867	(-0.528, 2.263)	0.223
	OR	95% CI	*P*	OR	95% CI	*P*	OR	95% CI	*P*
	**1.636**	**(1.070**,** 2.483)**	**0.022**	1.429	(0.923, 2.197)	0.106	1.409	(0.760, 2.592)	0.272
IEAA€	Beta	95% CI	*P*	Beta	95% CI	*P*	Beta	95% CI	*P*
	**1.607**	**(0.672**,** 2.542)**	**0.001**	**1.233**	**(0.279**,** 2.186)**	**0.011**	**1.523**	**(0.235**,** 2.812)**	**0.021**
	OR	95% CI	*P*	OR	95% CI	*P*	OR	95% CI	*P*
	**1.580**	**(1.197**,** 2.087)**	**0.001**	**1.448**	**(1.086**,** 1.931)**	**0.012**	**1.768**	**(1.165**,** 2.694)**	**0.008**
< Path 3>	OR	95% CI	*P*	OR	95% CI	*P*	OR	95% CI	*P*
Race	**1.473**	**(1.081**,** 2.021)**	**0.015**	1.338	(0.971, 1.856)	0.077	1.252	(0.799, 1.978)	0.330

AgeAccelDiff, epigenetic age acceleration as departure of DNAmAge from chronological age; CI, confidence interval; HYP, hypertension development; IEAA, intrinsic epigenetic age acceleration as residuals adjusted for cell composition; OR, odds ratio. Numbers in bold face are statistically significant

* Model 1: path 1 = HYP regressed on biological aging parameter; path 2 = biological aging parameters regressed on race (whites as reference); path 3 = HYP regressed on race (whites as reference)

** Model 2: Model 1 further included body mass index and waist-to-hip ratio as covariates

*** Model 3: Model 2 further included all other covariates (age, race, diabetes treated (pills or shots), high cholesterol requiring pills, fatty acids, whole fruits, and vegetables, and total score from Healthy Eating Index-2015, dietary alcohol, alcohol intake frequency, physical activity, years of regular smoking, oophorectomy history, hormone replacement therapy, ages at menopause and menarche, education, family income, any insurance and social support [except tested variable(s)])

¥ Results were similar when biological aging parameter was analyzed as a 10-year interval or the binary (negative vs. positive age acceleration)

€ Biological aging parameter was analyzed as either continuous or binary outcomes (negative vs. positive age acceleration)

### Analytic step 2 (Fig. 1B)

**Fig. 3:**
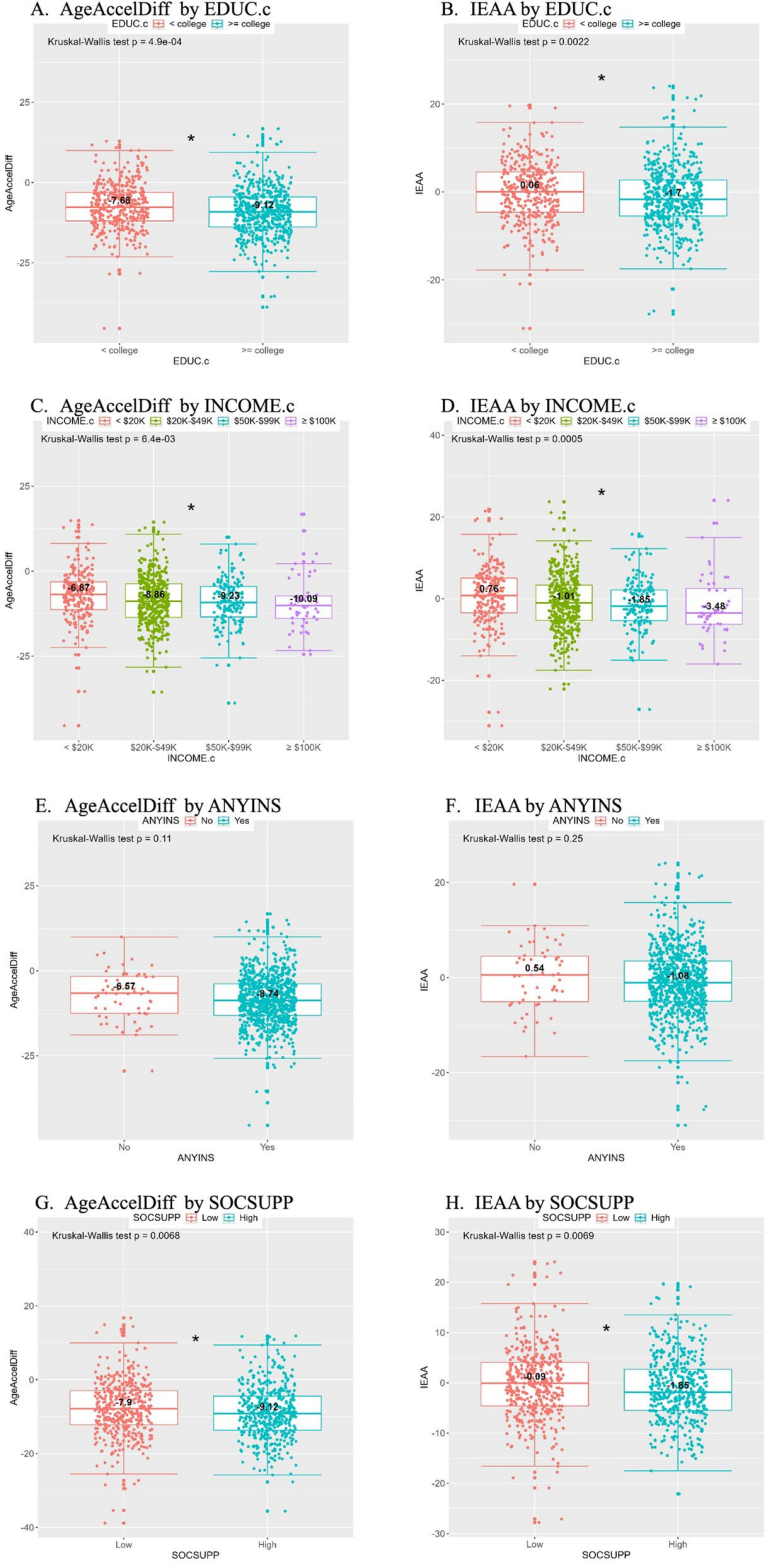
Analytic Step 2, path 1 in HYP data: distribution of AgeAccelDiff and IEAA by social determinants of health. (AgeAccelDiff, epigenetic age acceleration as departure of DNAmAge from chronological age; EDUC.c, education variable categorized; HYP, hypertension development; IEAA, intrinsic epigenetic age acceleration as residuals adjusted for cell composition; INCOME.c, family income variable categorized; ANYINS, any insurance variable categorized; SOCSUPP, social support construct categorized)

In Step 2 analysis, we examined two additional hypertension groups (HT.ch.n group for hypertension development and HT.ch.y group for hypertension improvement, both of which had been followed until AV3) in addition to the HYP and HT.ch.all groups. Across all four groups with different follow-up periods for hypertension outcomes, path 1 analyses showed consistent results: lower aging accel with greater education, greater income in a dose-response relationship, any medical insurance, and greater social support. Among those variables, education and income were statistically significant in a univariate manner across the groups (Figs. 3 and S4–S6). These patterns were similar between the two aging accel matrices (AgeAccelDiff and IEAA). In the path 2 analysis of racial difference in SDOH variables (Tables S6–S9), more AA women than whites tended to have no medical insurance, with statistical significance in Models 1 and/or 2 across the groups, and lower family income, with statistical significance in Model 3 for the HT.ch.n group. In contrast, AAs were more likely to have greater education levels than whites.

Further, we performed path 3 analysis by examining the mediation effect of SDOH variables, individually and jointly in AAs’ greater aging accel than whites’. Across all four groups with AgeAccelDiff outcomes, variability of mediation magnitudes was observed by SDOH type (Table 2). For instance, the effect of education on greater aging accel in AAs than in whites ranged from 4% to 47%, income from 4% to 43%, medical insurance from 2% to 14%, and social support from 14% to 22%; of note, those estimated mediation effects were not statistically significant. Similarly, the joint effect from combined SDOH variables on AAs’ greater aging accel ranged from 2% to 26%, but again, without statistical significance. For IEAA outcomes, the mediation effect sizes were relatively lower than those for AgeAccelDiff outcomes, ranging from 1% to 26%, and neither an individual nor joint effects of the SDOH variables substantially explained greater aging accel in AAs than in whites (Table 3). Our additional analyses with both aging accel matrices as binary outcomes provided similar results (Tables S10 and S11).

**Table 2:** Analytic Step 2, path 3: racial difference in biological aging parameter (AgeAccelDiff as a continuous outcome) and mediation effect of SDOH

	HYP				HT.ch.*n*				HT.ch.y					HT.ch.all			
	Beta	(95% CI)	*P*	%§, *P*	Beta	(95% CI)	*P*	%§, *P*	Beta	(95% CI)		*P*	%§, *P*	Beta	(95% CI)	*P*	%§, *P*
Race	0.667	(-0.666, 2.001)	0.326		1.550	(-0.965, 4.065)	0.226		1.471	(-0.680,	3.621)	0.179		**1.299**	**(0.250**,** 2.348)**	**0.015**	
Race plus education	0.981	(-0.361, 2.324)	0.151	47%, 0.104	1.853	(-0.704, 4.410)	0.155	19%, 0.744	1.544	(-0.621,	3.710)	0.162	5%, 0.412	**1.355**	**(0.302**,** 2.408)**	**0.012**	4%, 0.856
Race plus income	0.552	(-0.815, 1.919)	0.428	17%, 0.566	1.488	(-1.094, 4.070)	0.257	4%, 0.266	2.098	(-0.143,	4.340)	0.066	43%, 0.657	**1.231**	**(0.156**,** 2.305)**	**0.025**	4%, 0.254
Race plus insurance	0.684	(-0.650, 2.017)	0.315	2%, 0.261	1.776	(-0.738, 4.290)	0.165	14%, 0.474	1.528	(-0.656,	3.711)	0.170	4%, 0.551	**1.227**	**(0.173**,** 2.281)**	**0.023**	5%, 0.391
Race plus social-support	0.540	(-0.817, 1.898)	0.435	19%, 0.601	1.325	(-1.232, 3.881)	0.308	14%, 0.820	1.144	(-1.053,	3.342)	0.306	22%, 0.544	**1.107**	**(0.047**,** 2.168)**	**0.041**	15%, 0.855
Joint mediation¥	%	(95% CI)			%	(95% CI)			%	(95% CI)				%	(95% CI)		
	13%	(-0.347, 0.080)			5%	(-0.090, 0.138)			26%	(-0.843, 0.023)				2%	(-0.126, 0.060)		

AgeAccelDiff, epigenetic age acceleration as departure of DNAmAge from chronological age; HT.ch.all, hypertension status change with all outcomes at annual visit 3 (no development or improvement vs. development or no improvement); HT.ch.n, hypertension status change with two outcomes at annual visit 3 (no development vs. development); HT.ch.y, hypertension status change with two outcomes at annual visit 3 (improvement vs. no improvement); HYP, hypertension development; CI, confidence interval; SDOH, social determinants of health

¶ Results are presented from multivariate analysis including covariates (age, diabetes treated (pills or shots), high cholesterol requiring pills, fatty acids, whole fruits, and vegetables, and total score from Healthy Eating Index-2015, dietary alcohol, alcohol intake frequency, physical activity, years of regular smoking, oophorectomy history, hormone replacement therapy, ages at menopause and menarche, body mass index, and waist-to-hip ratio [except tested variable(s)])

§ The proportional effect of an individual SDOH was evaluated by the Sobel test

¥ The joint mediation effect combining all modeled SDOH was estimated and tested via a general multiple mediation analytic approach developed by Yu and Li [40, 41].

**Table 3:** Analytic Step 2, path 3: racial difference in biological aging parameter (IEAA as a continuous outcome) and mediation effect of SDOH

	HYP				HT.ch.*n*				HT.ch.y				HT.ch.all							
	Beta	(95% CI)	*P*	%§, *P*	Beta	(95% CI)	*P*	%§, *P*	Beta	(95% CI)	*P*	%§, *P*	Beta		(95% CI)		*P*		%§, *P*	
Race	**1.291**	**(0.061**,** 2.521)**	**0.040**		**1.687**	**(0.555**,** 2.820)**	**0.004**		**2.034**	**(0.102**,** 3.965)**	**0.039**		**1.852**		**(0.886**,** 2.819)**		**0.0002**			
Race plus education	**1.534**	**(0.295**,** 2.774)**	**0.015**	19%, 0.118	**1.726**	**(0.586**,** 2.865)**	**0.003**	2%, 0.744	**2.062**	**(0.121**,** 4.003)**	**0.037**	1%, 0.418	**1.880**		**(0.908**,** 2.852)**		**0.0001**		1%, 0.856	
Race plus income	**1.274**	**(0.017**,** 2.532)**	**0.047**	1%, 0.563	**1.448**	**(0.293**,** 2.603)**	**0.014**	14%, 0.201	**2.572**	**(0.567**,** 4.576)**	**0.012**	26%, 0.656	**1.812**		**(0.822**,** 2.802)**		**0.0003**		2%, 0.242	
Race plus insurance	**1.279**	**(0.049**,** 2.510)**	**0.042**	1%, 0.430	**1.597**	**(0.461**,** 2.734)**	**0.006**	5%, 0.743	**1.986**	**(0.032**,** 3.940)**	**0.046**	2%, 0.337	**1.776**		**(0.804**,** 2.747)**		**0.0003**		4%, 0.458	
Race plus social-support	1.205	(-0.047, 2.457)	0.059	7%, 0.599	**1.513**	**(0.370**,** 2.656)**	**0.010**	10%, 0.819	1.698	(-0.267, 3.663)	0.090	16%, 0.526	**1.654**		**(0.678**,** 2.631)**		**0.001**		11%, 0.855	
Joint mediation¥	%	(95% CI)			%	(95% CI)			%	(95% CI)			%		(95% CI)					
	14%	(-0.494, 0.050)			2%	(-0.120, 0.226)			8%	(-0.321, 0.061)			2%		(< 0.0001, 0.085)					

HT.ch.all, hypertension status change with all outcomes at annual visit 3 (no development or improvement vs. development or no improvement); HT.ch.n, hypertension status change with two outcomes at annual visit 3 (no development vs. development); HT.ch.y, hypertension status change with two outcomes at annual visit 3 (improvement vs. no improvement); HYP, hypertension development; CI, confidence interval; IEAA, intrinsic epigenetic age acceleration as residuals adjusted for cell composition; SDOH, social determinants of health

¶ Results are presented from multivariate analysis including covariates (age, diabetes treated (pills or shots), high cholesterol requiring pills, fatty acids, whole fruits, and vegetables, and total score from Healthy Eating Index-2015, dietary alcohol, alcohol intake frequency, physical activity, years of regular smoking, oophorectomy history, hormone replacement therapy, ages at menopause and menarche, body mass index, and waist-to-hip ratio [except tested variable(s)])

§ The proportional effect of an individual SDOH was evaluated by the Sobel test

¥ The joint mediation effect combining all modeled SDOH was estimated and tested via a general multiple mediation analytic approach developed by Yu and Li [40, 41].

### Analytic step 3 (Figs. 1C and S7)

In the mediation analyses of both HYP and HT.ch.all groups, the total and direct effects of race on hypertension outcomes were not much different from each other (Figure S7), suggesting a minimal influence of the modeled mediation effect on the greater incidence of hypertension in AA women. Accordingly, in the pathway of SDOH-induced aging accel, SDOH and aging accel parameters, both separately and jointly, mediated to a limited extent the racial difference in hypertension incidence at AV3 or during the mean 19-year follow-up period.

## Discussion

Substantial health disparities exist in epigenomic science for AAs. Thus, it is uncertain whether the existing epigenetic aging mechanisms observed in previous studies focusing on a majority of whites are applicable to AAs. Also, insufficient omics studies for women-specific hypertension phenomenon, especially among the aged, result in a severely limited scope of consequent findings and applications to the disease etiology and risk prediction. We addressed these in the study by evaluating Levine’s two biological aging accel matrices among older, postmenopausal women for their short-term and longer-term hypertension development and compared the rate of aging accel between AAs and whites, evaluating to what extent this disparity explained the greater incidence of hypertension in AAs than in whites.

Biological aging involves telomere shortening, genomic and epigenomic instabilities, cell communication changes, and cell homeostasis, all of which are frequently found in aging cells and tissues [8]. Telomere length has been associated with subclinical measures of functions or morphology in heart, kidney, and peripheral vasculature [42–44], but owing to variability of measurement methods and relatively weak association with aging-related conditions, its use is limited as an universal marker for biological aging [12]. Recently, epigenetic mechanisms, including DNAm alterations and microRNA, have been a topic of discussion in the aging process and aging-related diseases [10]. For instance, RNA modifications altering microRNA’s stability and function involve the pathogenesis of CVD [45, 46] and all-cause mortality [46]. In particular, dynamic DNAm has been known to outperform other aging biomarkers in its strong associations with aging and longevity [9]. Global DNA hypomethylation [47, 48] and local hypermethylation of the particular gene promoters [49, 50] were associated with the risk of CVD and aging-associated vascular diseases, including essential hypertension. One major challenge of hypertension’s primary prevention is the absence of early biomarkers to detect at-risk individuals. From this perspective, aging clocks measured via the levels of DNAm probes have increasingly been at the center of attention since deviation from chronological age, or “aging accel,” is well correlated with aging-related disorders. Indeed, epigenetic aging accel scales measured in blood are correlated with hypertension risk [36, 51–53], related death [13, 14], and its medications [54]. Our findings of greater aging accel in women who developed hypertension during a 3-year short-term and a mean 19-year longer-term follow-up are consistent with the previous findings, despite statistical insignificance.

In particular, AAs showed positive associations of epigenetic aging accel with BMI [55], brain vascular injury [56], hypertensive molecular phenotypes and target organ damage [57], and medications [58]. With these study findings, we established our hypothesis by demonstrating greater aging accel in AAs than whites and its contribution to the AAs’ high incidence of hypertension. However, our mediation test revealed minimal influence of AAs’ greater aging accel on the greater risk of hypertension development in AAs. This could be due to a lack of statistical power related to small variability of hypertension outcomes by the rate of aging accel, warranting a larger, independent validation study.

From a perspective of intersectional research, health inequity should be investigated at multiple axes, such as racial identity and social environment. SDOH are key to determining people’s health trajectory and central to studying the intersectional health disparity. SDOH, including an individual’s socioeconomic status (SES), shape the conditions of daily life and compromise an individual’s health by interacting with biological factors [59, 60], leading to a disproportionately high burden of disease such as cardiovascular risk factors [61] and hypertension [3, 23]. Selected SDOH, such as low sociodemographic factors, elevated aging accel as evidenced by lower education and socioeconomic levels were associated with increased aging accel [21, 22]; these correspond with our study findings from the Step 2, path 1 analysis. Having recognized a lack of information about the role of SDOH in mediating the racial difference in the incidence of hypertension via accelerated epigenetic aging, we examined the complex pathways measuring the extent to which SDOH-induced aging accel explained the greater incidence of hypertension in AAs than in whites. Our findings suggest an insignificant impact of SDOH and aging accel, individually and jointly, on the racial disparity in hypertension; this may result from insufficient variability of SDOH between races, resulting in a lack of statistical power. Further, current epigenetic clocks may not sufficiently capture the adverse social exposures on different biological aging processes by race. This calls for the development of a new epigenetic aging marker that is particularly responsive to a comprehensive set of SDOH, which can fully address aging processes owing to social exposures, so accurately evaluating the effect of adverse social exposures on differences in accelerated aging phenotypes between races.

By focusing on older women, our analyses accounted for a variety of women-specific reproductive histories and, with a large sample size, we followed hypertension outcomes during short- and longer-term periods via self-reporting as well as averages of BPs measured at different times, yielding relatively robust study findings. However, our study had several limitations. Modeled data did not contain the trajectories of aging accel scores in relation to hypertension incidence and progression. Removal of non-active women who had missing data on hypertension outcomes may result in our findings being affected by selection bias. Mutli-omics evaluations, including transcriptomics, proteomics, and metabolomics, can be used to systemically examine and enhance the resolution of exploring aging-related hypertension. In addition, our PBL-based epigenetic aging measures may limit our analysis of the association with hypertension as a systemic approach; however, aging accel matrices measured in PBLs have also been strongly correlated with those in multiple tissues, including spleen, bone marrow, lung, muscle, fat, kidney, and heart [62], suggesting comprehensive reflection across organs. Although our sensitivity analysis for the mediation tests to handle collider bias owing to BMI and WHR yielded results similar to those in the full adjustment models, the potential of collider stratification bias still remains, calling for caution in interpreting the results. Further, the change in the effect magnitude’s direction in the full adjustment model for the racial difference in DNAm age suggests that some post-race variables could have confound the aging pathway that is racially different. Finally, a comprehensive set of SDOH in multilevel pathways reflecting a hierarchical social structure may reveal more pronounced socio-biological aging processes in hypertension outcomes.

## Conclusions

In conclusion, we found greater biological aging associated with hypertension outcomes and greater aging accel in AAs, which was partially mediated via adverse social exposures. Further SDOH-induced aging accel mediated AAs’ greater incidence of hypertension to a small extent. Our findings warrant further validation in independent multi-omics, epigenetic trajectory studies. Our study contributes to a better understanding of the socio-biological hypertensive pathways shared by aging processes, which may inform risk stratification in the elderly and preventive practices to address SDOH that affect downstream aging pathways for hypertension prevention and racial disparity.

## Supplementary Information

Below is the link to the electronic supplementary material.

Supplementary Material 1.

## Data Availability

The data that support the findings of this study are available in accordance with policies developed by the NHLBI and WHI in order to protect sensitive participant information and approved by the Fred Hutchinson Cancer Research Center, which currently serves as the IRB of record for the WHI. Data requests may be made by emailing [helpdesk@WHI.org](mailto: helpdesk@WHI.org) .

## Abbreviations

AAs: African Americans
AV: Annual visit
Aging accel: Biological aging acceleration
BMI: Body mass index
BP: Blood pressure
CpGs: CpG dinucleotides
DNAm: DNA methylation
HEI-2015: Healthy eating index-2015
HYP: Hypertension
IEAA: Intrinsic epigenetic age acceleration
PBL: Peripheral blood lymphocyte
SDOH: Social determinants of health
SES: Socioeconomic status
WHI: Women’s health initiative
WHI-dbGaP: Women’s Health initiative database for genotypes and phenotypes
WHR: Waist-to-hip ratio
