# Supplementary material for: Hypertension and epigenetic aging: socio-biological pathways of health disparity
Source: Clin Epigenetics. 2026 Apr 4;18:98. doi: 10.1186/s13148-026-02117-w (PMC13214139; doi:10.1186/s13148-026-02117-w)

Table S1. Participants' characteristics**

Characteristic	Whites (n = 629)		AAs (n = 297)	
	n	(%)	n	(%)
**Age in years, median (range)***	68	(50 – 80)	62	(50 – 79)
**BMI in kg/m^2, median (range)***	28.39	(17.31 – 58.27)	30.86	(17.21 – 60.79)
**Waist-to-hip ratio, median (range)**	0.826	(0.566 – 1.673)	0.833	(0.332 – 1.895)
**METs·hour·week^{-1}¶***	7.0	(0.00 – 87.5)	3.7	(0.00 – 93.0)
**Years of regular smoking***				
**Never smoker**	527	(54.4)	319	(49.3)
**<5**	108	(11.2)	107	(16.5)
**5-<20**	108	(11.2)	119	(18.4)
**>=20**	225	(23.2)	102	(15.8)
**Dietary alcohol per day in g, median (range)***	0.8	(0.00 – 191.9)	0.03	(0.00 – 202.3)
**Frequency of alcohol consumption***				
**Non drinker**	128	(12.9)	128	(19.1)
**Past drinker**	175	(17.6)	229	(34.1)
**<1 drink per month**	144	(14.5)	73	(10.9)
**<1 drink per week**	188	(19.0)	137	(20.4)
**1 to <7 drinks per week**	234	(23.6)	75	(11.2)
**7+ drinks per week**	123	(12.4)	29	(4.3)
**HEI-2015 total score, median (range)***	65.3	(33.8 – 90.8)	63.0	(31.6 – 93.2)
**HEI-2015 score for fatty acids, median (range)***	4.2	(0 – 10)	5.5	(0 – 10)
**HEI-2015 score for total vegetables, median (range)***	4.2	(0.8 – 5.0)	3.5	(0.1 – 5.0)
**HEI-2015 score for whole fruits, median (range)***	5.0	(0.1 – 5.0)	5.0	(0.005 – 5.0)
**Diabetes treated (pills or shots)***				
**No**	936	(93.8)	565	(83.6)
**Yes**	62	(6.2)	111	(16.4)
**High cholesterol requiring pills ever**				
**No**	857	(85.9)	563	(83.3)
**Yes**	141	(14.1)	113	(16.7)
**Hypertension development***				
**No**	204	(32.4)	73	(24.6)
**Yes**	425	(67.6)	224	(75.4)
**Age at menarche in years, median (range)**	13	(≤ 9 – ≥ 17)	13	(≤ 9 – ≥ 17)
**Age at menopause in years, median (range)***	50	(25 – 60)	48	(25 – 60)
**Oophorectomy***				
**No**	709	(72.9)	385	(58.7)
**Yes, one , unknown, and part taken out**	82	(8.4)	126	(19.2)
**Yes, both were taken out**	182	(18.7)	145	(22.1)
**Exogenous estrogen use (E only use)**				
**Never use**	715	(71.6)	466	(68.9)
**< 5 Years**	166	(16.6)	109	(16.1)
**5 to < 10 Years**	47	(4.7)	30	(4.4)
**10 + Years**	70	(7.0)	71	(10.5)
**Exogenous estrogen use (E + P use)**				

**Never use**	915	(91.7)	607	(89.9)
**< 5 Years**	58	(5.8)	43	(6.4)
**5 to < 10 Years**	14	(1.4)	15	(2.2)
**10 + Years**	11	(1.1)	10	(1.5)
**SDOH**				
**Education**				
**< College**	429	(43.2)	286	(42.8)
**≥ College**	563	(56.8)	382	(57.2)
**Family income***				
**< $20,000**	245	(26.0)	204	(32.0)
**$20,000-$49,999**	479	(50.7)	283	(44.4)
**$50,000-$99,999**	180	(19.1)	119	(18.7)
**≥ $100,000**	40	(4.2)	32	(5.0)
**Any medical insurance***				
**No**	41	(4.1)	57	(8.6)
**Yes**	950	(95.9)	608	(91.4)
**Social support construct, median (range)**	37	(10 – 45)	37	(9 – 45)

AAs, African Americans; BMI, body mass index; E, estrogen; E+P, estrogen plus progestin; HEI-2015, Healthy Eating Index-2015; MET, metabolic equivalent; SDOH, social determinants of health.

* $P < 0.05$, chi-squared or Wilcoxon's rank-sum test.

** Participants (n = 926) with available long-term (a mean 19-year) hypertension outcome are included.

¶ Physical activity was estimated from recreational physical activity combining walking and mild, moderate, and strenuous physical activity. Each activity was assigned a MET value corresponding to intensity; the total MET·hours·week^{-1} was calculated by multiplying the MET level for the activity by the hours exercised per week and summing the values for all activities.

Table S2. Social determinants of health variables and their questions

S2A. Education

What is the highest grade in school you finished? (Mark one.)	
**Value**	**Description**
1	Didn't go to school
2	Grade school (1-4 years)
3	Grade school (5-8 years)
4	Some high school (9-11 years)
5	High school diploma or GED
6	Vocational or training school
7	Some college or Associate Degree
8	College graduate or Baccalaureate Degree
9	Some post-graduate or professional
10	Master's Degree
11	Doctoral Degree (Ph.D,M.D.,J.D.,etc.)

S2B. Family income

What was the total family income (before taxes) from all sources within your household in the last year?	
**Value**	**Description**
1	Less than $10,000
2	$10,000 to $19,999
3	$20,000 to $34,999
4	$35,000 to $49,999
5	$50,000 to $74,999
6	$75,000 to $99,999
7	$100,000 to $149,999
8	$150,000 or more
9	Don't know

S2C. Any insurance

Indicator for whether the participant has any medical insurance	
**Value**	**Description**
0	No
1	Yes

S2D. Social support

People sometimes look to others for help, friendship, or other types of support. Next are some questions about the support that you have. How often is each of the following kinds of support available to you if you need it? (Mark one on each line)					
**Questions**	**None of the time**	**A little of the time**	**Some of the time**	**Most of the time**	**All of the time**
1. Someone you can count on to listen to you when you need to talk					
2. Someone to give you good advice about a problem					

3. Someone to take you to the doctor if you need it					
4. Some to have a good time with					
5. Someone to help you understand a problem when you need it					
6. Someone to help with daily chores if you are sick					
7. Someone to share your most private worries and fears					
8. Someone to do something fun with					
9. Someone to love you and make you feel wanted					

Table S3. Definition of hypertension groups at AV3

	HT.ch.n	N (%)	HT.ch.y	n (%)	HT.ch.all group	n (%)
0 (reference)	no hypertension development	855 (82)	normal blood pressure in those with baseline hypertension	250 (54)	no hypertension development or improvement	1,105 (73)
1	hypertension development	184 (18)	continued high blood pressure in those with baseline hypertension	216 (46)	hypertension development or no improvement	400 (27)
Total sample size		1,039 (100)		466 (100)		1,505 (100)

Table S4. Analytic step 1 with 3 pathways: association of biologic aging parameters (DNAmAge, AgeAccelDiff, and IEAA) with HT.ch.all (path 1) and racial difference in biologic aging parameter (path 2) and HT.ch.all (path 3).

	**Model 1***			**Model 2****			**Model 3*****		
**<Pathway 1>**	**OR**	**95% CI**	***P***	**OR**	**95% CI**	***P***	**OR**	**95% CI**	***P***
**DNAmAge¶**	**1.016**	**(1.004, 1.028)**	**0.008**	**1.015**	**(1.004, 1.027)**	**0.010**	1.002	(0.984, 1.021)	0.830
**AgeAccelDiff¥**	1.009	(0.994, 1.025)	0.247	1.004	(0.989, 1.020)	0.580	1.002	(0.984, 1.021)	0.830
**IEAA¥**	1.011	(0.994, 1.028)	0.194	1.005	(0.989, 1.022)	0.539	0.999	(0.979, 1.019)	0.929
**<Pathway 2>**	**Beta**	**95% CI**	***P***	**Beta**	**95% CI**	***P***	**Beta**	**95% CI**	***P***
**DNAmAge**	**-4.005**	**(-5.028, -2.982)**	**2.80E-14**	**-4.023**	**(-5.073, -2.972)**	**9.97E-14**	**1.408**	**(0.310, 2.506)**	**0.012**
**AgeAccelDiff€**	**Beta**	**95% CI**	***P***	**Beta**	**95% CI**	***P***	**Beta**	**95% CI**	***P***
	**1.578**	**(0.802, 2.353)**	**6.94E-05**	**1.143**	**(0.351, 1.935)**	**0.005**	**1.408**	**(0.310, 2.506)**	**0.012**
	**OR**	**95% CI**	***P***	**OR**	**95% CI**	***P***	**OR**	**95% CI**	***P***
	**1.789**	**(1.338, 2.395)**	**8.86E-05**	**1.602**	**(1.185, 2.165)**	**0.002**	**1.613**	**(1.059, 2.456)**	**0.026**
**IEAA€**	**Beta**	**95% CI**	***P***	**Beta**	**95% CI**	***P***	**Beta**	**95% CI**	***P***
	**1.964**	**(1.247, 2.681)**	**8.98E-08**	**1.566**	**(0.836, 2.297)**	**2.73E-05**	**1.918**	**(0.905, 2.931)**	**0.0002**
	**OR**	**95% CI**	***P***	**OR**	**95% CI**	***P***	**OR**	**95% CI**	***P***
	**1.562**	**(1.268, 1.925)**	**2.79E-05**	**1.421**	**(1.145, 1.764)**	**0.001**	**1.633**	**(1.205, 2.216)**	**0.002**
**<Pathway 3>**	**OR**	**95% CI**	***P***	**OR**	**95% CI**	***P***	**OR**	**95% CI**	***P***
**Race**	1.045	(0.826, 1.319)	0.715	0.962	(0.754, 1.225)	0.755	1.050	(0.747, 1.474)	0.778

AgeAccelDiff, epigenetic age acceleration as departure of DNAmAge from chronologic age; CI, confidence interval; DNAmAge, DNA methylation–based marker of aging; HT.ch.all, hypertension status change with all outcomes at annual visit 3 (no development or improvement [reference] vs. development or no improvement); IEAA, intrinsic epigenetic age acceleration as residuals adjusted for cell composition; OR, odds ratio. Numbers in bold face are statistically significant.

* Model 1: pathway 1 = HT.ch.all regressed on biologic aging parameter; pathway 2 = biologic aging parameters regressed on race (whites as reference); pathway 3 = HT.ch.all regressed on race (white as reference).

** Model 2: Model 1 further included body mass index, waist-to-hip ratio as a covariate.

*** Model 3: Model 2 further included all other covariates (age, race, diabetes treated (pills or shots), high cholesterol requiring pills, fatty acids, whole fruits, and vegetables, and total score from Healthy Eating Index-2015, dietary alcohol, alcohol intake frequency,

physical activity, years of regular smoking, oophorectomy history, hormone replacement therapy, ages of menopause and menarche, education, family income, any insurance and social support [except tested variable(s)]).
¶ Results were similar when biologic aging parameter was analyzed via a 10-year interval (negative vs. positive age acceleration).
¥ Results were similar when biologic aging parameter was analyzed via a 10-year interval or binary (negative vs. positive age acceleration).
€ Biologic aging parameter was analyzed as either continuous or binary outcomes (negative vs. positive age acceleration).

Table S5. Analytic step 1 with 3 pathways: association of DNAmAge with HYP (path 1) and racial difference in DNAmAge (path 2) and HYP (path 3).

	**Model 1***				**Model 2****				**Model 3*****			
**<Pathway 1>**	**OR**	**95% CI**		***P***	**OR**	**95% CI**		***P***	**OR**	**95% CI**		***P***
**DNAmAge¥**	0.997	0.983	1.011	0.651	0.996	0.982	1.010	0.590	1.006	0.982	1.031	0.627
**<Pathway 2>**	**Beta**	**95% CI**		***P***	**Beta**	**95% CI**		***P***	**Beta**	**95% CI**		***P***
**DNAmAge**	**-4.560**	**-5.904**	**-3.215**	**4.9E-11**	**-4.634**	**-6.011**	**-3.256**	**7.0E-11**	0.867	-0.528	2.263	0.223

CI, confidence interval; DNAmAge, DNA methylation–based marker of aging; HYP, hypertension development; IEAA, intrinsic epigenetic age acceleration as residuals adjusted for cell composition; OR, odds ratio. Numbers in bold face are statistically significant.

* Model 1: pathway 1 = HYP regressed on DNAmAge; pathway 2 = DNAmAge regressed on race (whites as reference).

** Model 2: Model 1 further included body mass index, waist-to-hip ratio as a covariate.

*** Model 3: Model 2 further included all other covariates (age, race, diabetes treated (pills or shots), high cholesterol requiring pills, fatty acids, whole fruits, and vegetables, and total score from Healthy Eating Index-2015, dietary alcohol, alcohol intake frequency, physical activity, years of regular smoking, oophorectomy history, hormone replacement therapy, ages of menopause and menarche, education, family income, any insurance and social support [except tested variable(s)]).

¥ Results were similar when DNAmAge parameter was analyzed via a 10-year interval.

Table S6. Analytic step 2, pathway 2 in HYP data: association of race with social determinants of health.

SDOH	Model 1*			Model 2**			Model 3***		
	**OR**	**95% CI**	***P***	**OR**	**95% CI**	***P***	**OR**	**95% CI**	***P***
**Education**	1.307	(0.985, 1.741)	0.065	1.318	(0.984, 1.771)	0.065	**2.236**	**(1.439, 3.510)**	**0.0004**
**Family income**	0.897	(0.685, 1.173)	0.427	0.971	(0.736, 1.279)	0.833	0.789	(0.537, 1.159)	0.228
**Any insurance**	**0.533**	**(0.313, 0.912)**	**0.020**	0.600	(0.347, 1.044)	0.067	1.229	(0.452, 3.493)	0.691
	**Beta**	**95% CI**	***P***	**Beta**	**95% CI**	***P***	**Beta**	**95% CI**	***P***
**Social support**	-0.316	(-1.425, 0.793)	0.576	-0.097	(-1.235, 1.041)	0.868	0.281	(-1.150, 1.712)	0.700

CI, confidence interval; HYP, hypertension development; OR, odds ratio; SDOH, social determinant of health. Numbers in bold face are statistically significant.

* Model 1: pathway 2 = social determinants of health regressed on race (white as reference).

** Model 2: Model 1 further included body mass index, waist-to-hip ratio as a covariate.

*** Model 3: Model 2 further included all other covariates (age, race, diabetes treated (pills or shots), high cholesterol requiring pills, fatty acids, whole fruits, and vegetables, and total score from Healthy Eating Index-2015, dietary alcohol, alcohol intake frequency, physical activity, years of regular smoking, oophorectomy history, hormone replacement therapy, ages of menopause and menarche, education, family income, any insurance and social support [except tested variable(s)]).

Table S7. Analytic step 2, pathway 2 in in HT.ch.n data: association of race with social determinants of health.

**SDOH**	**Model 1***				**Model 2****				**Model 3*****			
	**OR**	**95% CI**		***P***	**OR**	**95% CI**		***P***	**OR**	**95% CI**		***P***
**Education**	0.958	0.742	1.238	0.741	0.988	0.758	1.288	0.928	1.478	0.996	2.203	0.053
**Family income**	0.795	0.624	1.011	0.062	0.889	0.693	1.138	0.350	**0.708**	**0.503**	**0.997**	**0.048**
**Any insurance**	**0.587**	**0.349**	**0.989**	**0.044**	0.668	0.390	1.147	0.141	1.298	0.519	3.312	0.579
	**Beta**	**95% CI**		***P***	**Beta**	**95% CI**		***P***	**Beta**	**95% CI**		***P***
**Social support**	0.118	-0.884	1.120	0.817	0.353	-0.678	1.384	0.502	0.994	-0.306	2.294	0.134

CI, confidence interval; HT.ch.n, hypertension status change with two outcomes at annual visit 3 (no development vs. development); OR, odds ratio; SDOH, social determinant of health. Numbers in bold face are statistically significant.

* Model 1: pathway 2 = social determinants of health regressed on race (white as reference).

** Model 2: Model 1 further included body mass index, waist-to-hip ratio as a covariate.

*** Model 3: Model 2 further included all other covariates (age, race, diabetes treated (pills or shots), high cholesterol requiring pills, fatty acids, whole fruits, and vegetables, and total score from Healthy Eating Index-2015, dietary alcohol, alcohol intake frequency, physical activity, years of regular smoking, oophorectomy history, hormone replacement therapy, ages of menopause and menarche, education, family income, any insurance and social support [except tested variable(s)]).

Table S8. Analytic step 2, pathway 2 in in HT.ch.y data: association of race with social determinants of health.

SDOH	Model 1*			Model 2**			Model 3***		
	OR	95% CI	*P*	OR	95% CI	*P*	OR	95% CI	*P*
**Education**	1.187	(0.818, 1.724)	0.367	1.223	(0.834, 1.798)	0.304	**1.970**	**(1.065, 3.700)**	**0.032**
**Family income**	1.056	(0.735, 1.520)	0.767	1.104	(0.759, 1.606)	0.603	1.417	(0.820, 2.456)	0.212
**Any insurance**	**0.343**	**(0.128, 0.842)**	**0.024**	**0.372**	**(0.136, 0.936)**	**0.041**	0.495	(0.061, 3.121)	0.478
	**Beta**	**95% CI**	***P***	**Beta**	**95% CI**	***P***	**Beta**	**95% CI**	***P***
**Social support**	-0.524	(-1.929, 0.882)	0.464	-0.255	(-1.704, 1.193)	0.729	-0.428	(-2.402, 1.547)	0.670

CI, confidence interval; HT.ch.y, hypertension status change with two outcomes at annual visit 3 (improvement vs. no improvement); OR, odds ratio; SDOH, social determinant of health. Numbers in bold face are statistically significant.

* Model 1: pathway 2 = social determinants of health regressed on race (white as reference).

** Model 2: Model 1 further included body mass index, waist-to-hip ratio as a covariate.

*** Model 3: Model 2 further included all other covariates (age, race, diabetes treated (pills or shots), high cholesterol requiring pills, fatty acids, whole fruits, and vegetables, and total score from Healthy Eating Index-2015, dietary alcohol, alcohol intake frequency, physical activity, years of regular smoking, oophorectomy history, hormone replacement therapy, ages of menopause and menarche, education, family income, any insurance and social support [except tested variable(s)]).

Table S9. Analytic step 2, pathway 2 in in HT.ch.all data: association of race with social determinants of health.

SDOH	Model 1*			Model 2**			Model 3***		
	**OR**	**95% CI**	***P***	**OR**	**95% CI**	***P***	**OR**	**95% CI**	***P***
**Education**	1.020	(0.826, 1.259)	0.856	1.051	(0.845, 1.307)	0.656	**1.596**	**(1.153, 2.218)**	**0.005**
**Family income**	0.859	(0.703, 1.050)	0.138	0.946	(0.770, 1.162)	0.595	0.847	(0.637, 1.127)	0.255
**Any insurance**	**0.520**	**(0.331, 0.813)**	**0.004**	**0.588**	**(0.369, 0.932)**	**0.024**	0.974	(0.465, 2.038)	0.945
	**Beta**	**95% CI**	***P***	**Beta**	**95% CI**	***P***	**Beta**	**95% CI**	***P***
**Social support**	-0.076	(-0.892, 0.739)	0.854	0.163	(-0.676, 1.001)	0.703	0.634	(-0.441, 1.709)	0.248

CI, confidence interval; HT.ch.all, hypertension status change with all outcomes at annual visit 3 (no development or improvement vs. development or no improvement); OR, odds ratio; SDOH, social determinants of health. Numbers in bold face are statistically significant.
* Model 1: pathway 2 = social determinant of health regressed on race (white as reference).
** Model 2: Model 1 further included body mass index, waist-to-hip ratio as a covariate.
*** Model 3: Model 2 further included all other covariates (age, race, diabetes treated (pills or shots), high cholesterol requiring pills, fatty acids, whole fruits, and vegetables, and total score from Healthy Eating Index-2015, dietary alcohol, alcohol intake frequency, physical activity, years of regular smoking, oophorectomy history, hormone replacement therapy, ages of menopause and menarche, education, family income, any insurance and social support [except tested variable(s)]).

Table S10. Analytic Step 2, path 3: racial difference in biologic aging parameter (AgeAccelDiff as a binary outcome) and mediation effect of SDOH

	**HYP**				**HT.ch.n**				**HT.ch.y**				**HT.ch.all**			
	**OR¶**	**(95% CI)**	***P***	**%§, *P***	**OR ¶**	**(95% CI)**	***P***	**%§, *P***	**OR ¶**	**(95% CI)**	***P***	**%§, *P***	**OR ¶**	**(95% CI)**	***P***	**%§, *P***
**Race**	1.199	0.671 2.120	0.535		1.570	0.526 4.585	0.411		1.365	0.684 2.726	0.376		**1.618**	**1.092 2.398**	**0.016**	
**Race plus education**	1.394	0.772 2.496	0.266	16%, 0.104	1.966	0.622 6.122	0.243	25%, 0.744	1.432	0.714 2.880	0.311	5%, 0.412	**1.701**	**1.144 2.532**	**0.009**	5%, 0.856
**Race plus income**	1.187	0.651 2.139	0.571	1%, 0.566	1.618	0.535 4.806	0.387	3%, 0.266	1.508	0.733 3.116	0.264	10%, 0.657	**1.506**	**1.004 2.259**	**0.048**	7%, 0.254
**Race plus insurance**	1.231	0.685 2.187	0.482	3%, 0.261	1.952	0.640 5.904	0.234	24%, 0.474	1.441	0.713 2.921	0.308	5%, 0.551	**1.591**	**1.068 2.373**	**0.022**	2%, 0.391
**Race plus social-support**	1.223	0.684 2.163	0.493	2%, 0.601	1.582	0.524 4.674	0.408	1%, 0.820	1.228	0.606 2.481	0.567	10%, 0.544	1.446	0.966 2.163	0.073	11%, 0.855
**Joint mediation¥**	**%**	**(95% CI)**			**%**	**(95% CI)**			**%**	**(95% CI)**			**%**	**(95% CI)**		
	13%	(-0.347, 0.080)			5%	(-0.090, 0.138)			26%	(-0.843, 0.023)			2%	(-0.126, 0.060)		

AgeAccelDiff, epigenetic age acceleration as departure of DNAmAge from chronological age; CI, confidence interval; HT.ch.all, hypertension status change with all outcomes at annual visit 3 (no development or improvement vs. development or no improvement); HT.ch.n, hypertension status change with two outcomes at annual visit 3 (no development vs. development); HT.ch.y, hypertension status change with two outcomes at annual visit 3 (improvement vs. no improvement); HYP, hypertension development; OR, odds ratio; SDOH, social determinants of health.

¶ Results are presented from multivariate analysis including covariates (age, diabetes treated (pills or shots), high cholesterol requiring pills, fatty acids, whole fruits, and vegetables, and total score from Healthy Eating Index-2015, dietary alcohol, alcohol intake frequency, physical activity, years of regular smoking, oophorectomy history, hormone replacement therapy, ages at menopause and menarche, body mass index, and waist-to-hip ratio [except tested variable(s)]).

§ The proportional effect of an individual SDOH was evaluated by the Sobel test.

¥ The joint mediation effect combining all modeled SDOH was estimated and tested via a general multiple mediation analytic approach developed by Yu and Li.[40,41]

Table S11. Analytic Step 2, path 3: racial difference in biological aging parameter (IEAA as a binary outcome) and mediation effect of SDOH

	**HYP**				**HT.ch.n**				**HT.ch.y**				**HT.ch.all**			
	**OR¶**	**(95% CI)**	***P***	**%§, *P***	**OR ¶**	**(95% CI)**	***P***	**%§, *P***	**OR ¶**	**(95% CI)**	***P***	**%§, *P***	**OR ¶**	**(95% CI)**	***P***	**%§, *P***
**Race**	**1.743**	**(1.178, 2.589)**	**0.006**		**1.604**	**(1.128, 2.285)**	**0.009**		1.629	(0.953, 2.807)	0.076		**1.639**	**(1.230, 2.190)**	**0.001**	
**Race plus education**	**1.846**	**(1.232, 2.778)**	**0.003**	6%, 0.118	**1.590**	**(1.115, 2.273)**	**0.011**	1%, 0.744	1.646	(0.957, 2.857)	0.073	1%, 0.418	**1.631**	**(1.220, 2.184)**	**0.001**	0.5%, 0.856
**Race plus income**	**1.680**	**(1.123, 2.525)**	**0.012**	4%, 0.563	**1.549**	**(1.081, 2.224)**	**0.017**	3%, 0.201	1.793	(1.020, 3.188)	0.044	10%, 0.656	**1.629**	**(1.212, 2.193)**	**0.001**	1%, 0.242
**Race plus insurance**	**1.738**	**(1.173, 2.585)**	**0.006**	0.3%, 0.430	**1.576**	**(1.107, 2.250)**	**0.012**	2%, 0.743	1.630	(0.943, 2.842)	0.082	0.1%, 0.337	**1.615**	**(1.209, 2.162)**	**0.001**	1%, 0.458
**Race plus social-support**	**1.679**	**(1.131, 2.501)**	**0.010**	4%, 0.599	**1.546**	**(1.080, 2.217)**	**0.017**	3%, 0.819	1.510	(0.878, 2.614)	0.138	7%, 0.526	**1.577**	**(1.178, 2.114)**	**0.002**	4%, 0.855
**Joint mediation¥**	**%**	**(95% CI)**			**%**	**(95% CI)**			**%**	**(95% CI)**			**%**	**(95% CI)**		
	14%	(-0.494, 0.050)			2%	(-0.120, 0.226)			8%	(-0.321, 0.061)			2%	(< 0.0001, 0.085)		

CI, confidence interval; HT.ch.all, hypertension status change with all outcomes at annual visit 3 (no development or improvement vs. development or no improvement); HT.ch.n, hypertension status change with two outcomes at annual visit 3 (no development vs. development); HT.ch.y, hypertension status change with two outcomes at annual visit 3 (improvement vs. no improvement); HYP, hypertension development; IEAA, intrinsic epigenetic age acceleration as residuals adjusted for cell composition; OR, odds ratio; SDOH, social determinants of health.

¶ Results are presented from multivariate analysis including covariates (age, diabetes treated (pills or shots), high cholesterol requiring pills, fatty acids, whole fruits, and vegetables, and total score from Healthy Eating Index-2015, dietary alcohol, alcohol intake frequency, physical activity, years of regular smoking, oophorectomy history, hormone replacement therapy, ages at menopause and menarche, body mass index, and waist-to-hip ratio [except tested variable(s)]).

§ The proportional effect of an individual SDOH was evaluated by the Sobel test.

¥ The joint mediation effect combining all modeled SDOH was estimated and tested via a general multiple mediation analytic approach developed by Yu and Li.[40,41]

Figure S1. Flowchart for the selection of the study population (AAs, African American; AV3, annual visit 3; HYP, hypertension)

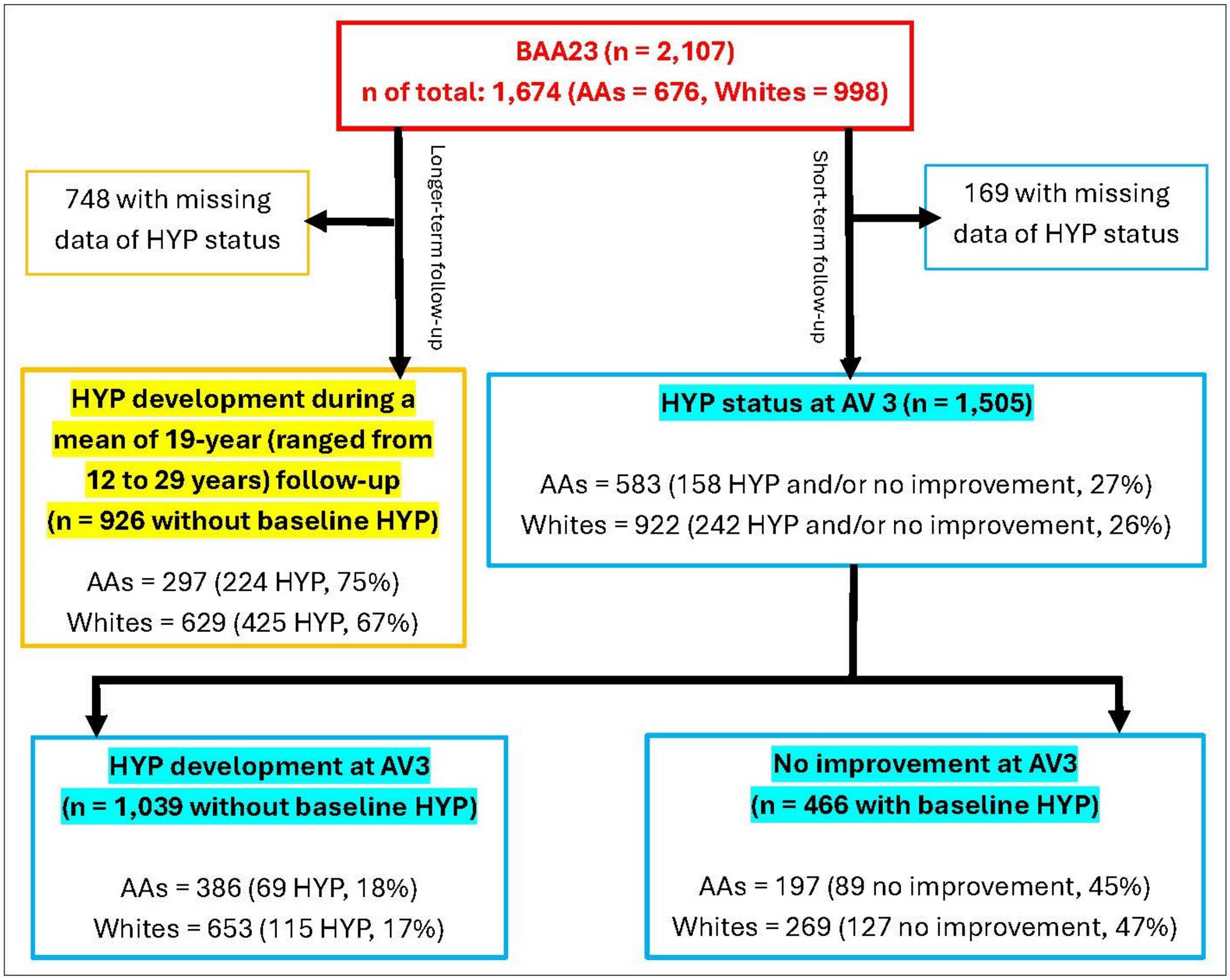

Figure S2. Analytic step 1: distribution of DNAmAge, AgeAccelDiff, and IEAA by HT.ch.all and race. A, B, and C, pathway 1 of the analytic step; D, E, and F, pathway 2 of the analytic step. (AgeAccelDiff, epigenetic age acceleration as departure of DNAmAge from chronologic age; B, blacks; DNAmAge, DNA methylation–based marker of aging; HT.ch.all, hypertension status change with all outcomes at annual visit 3 (no development or improvement [No] vs. development or no improvement [Yes]); IEAA, intrinsic epigenetic age acceleration as residuals adjusted for cell composition; W, whites.)

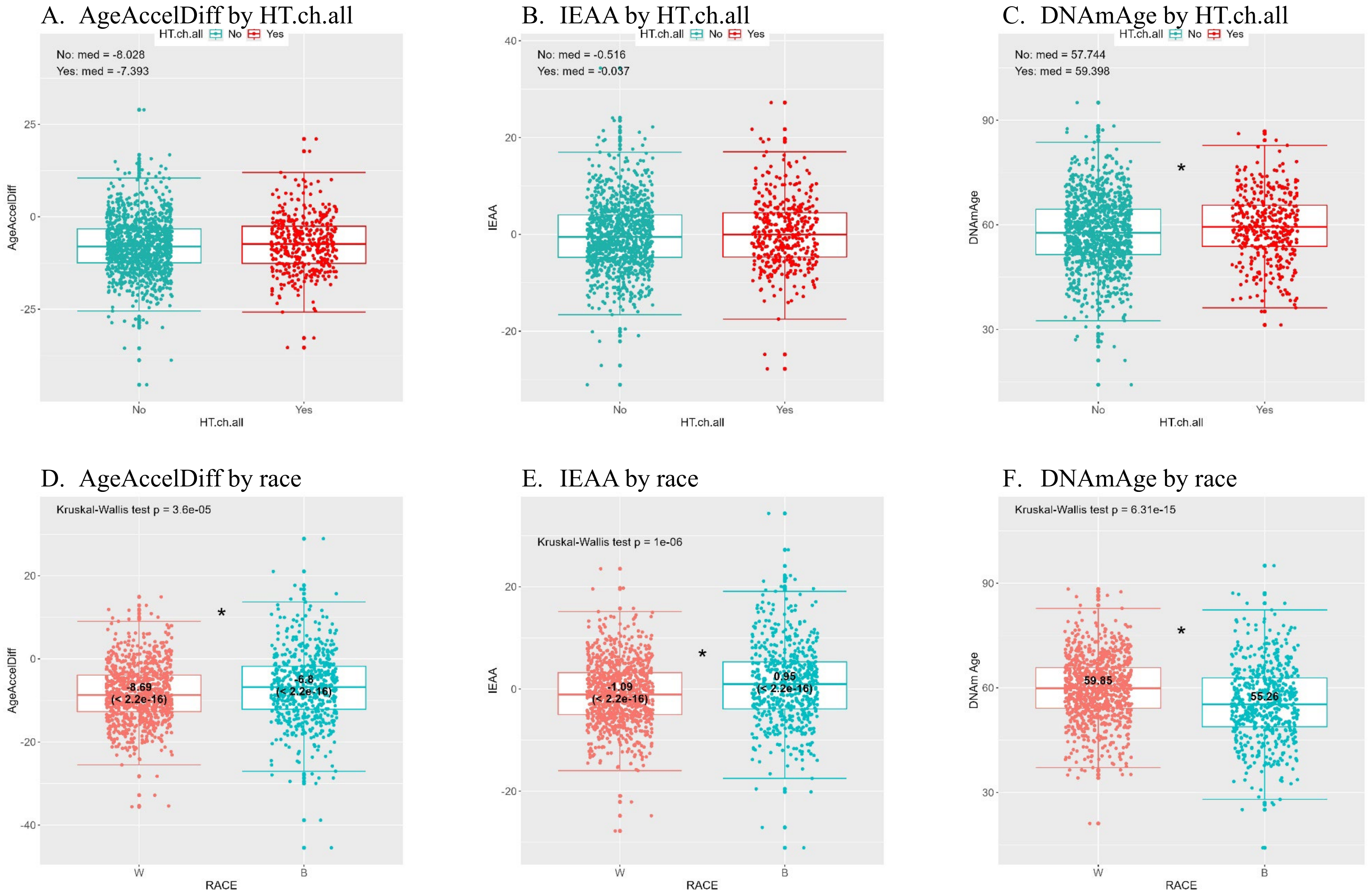

Figure S3. Analytic step 1: distribution of DNAmAge by HYP and race. A, pathway 1 of the analytic step; B, pathway 2 of the analytic step. (B, blacks; DNAmAge, DNA methylation–based marker of aging; HYP, hypertension development; W, whites.)

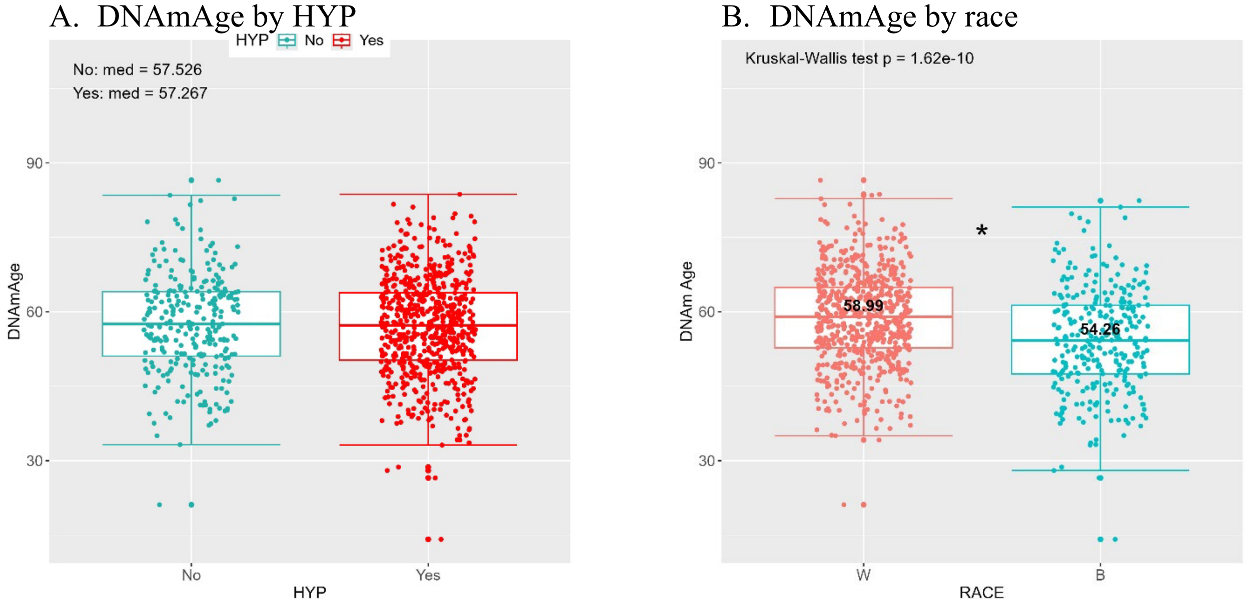

Figure S4. Analytic step 2, pathway 1 in HT.ch.n data: distribution of AgeAccelDiff and IEAA by social determinants of health. (AgeAccelDiff, epigenetic age acceleration as departure of DNAmAge from chronologic age; ANYINS, any insurance variable categorized; EDUC.c, education variable categorized; HT.ch.n, hypertension status change with two outcomes at annual visit 3 (no development vs. development); IEAA, intrinsic epigenetic age acceleration as residuals adjusted for cell composition; INCOME.c, family income variable categorized; SOCSUPP, social support construct categorized.)

A. AgeAccelDiff by EDUC.c

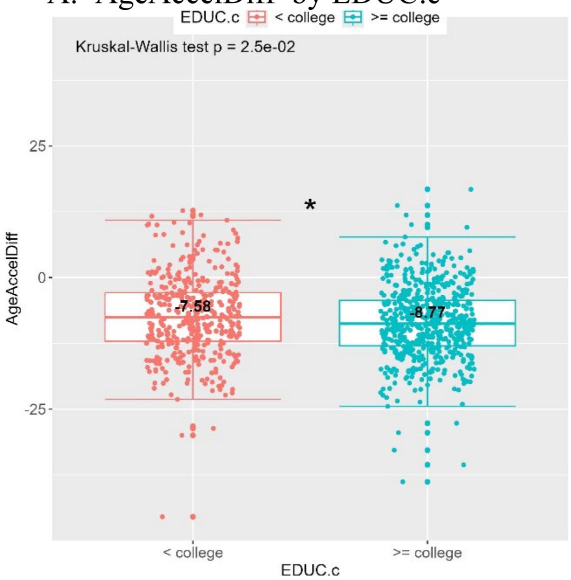

B. IEAA by EDUC.c

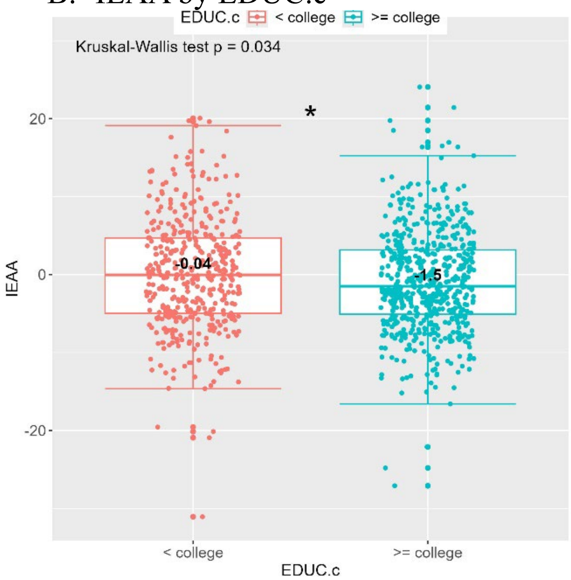

C. AgeAccelDiff by INCOME.c

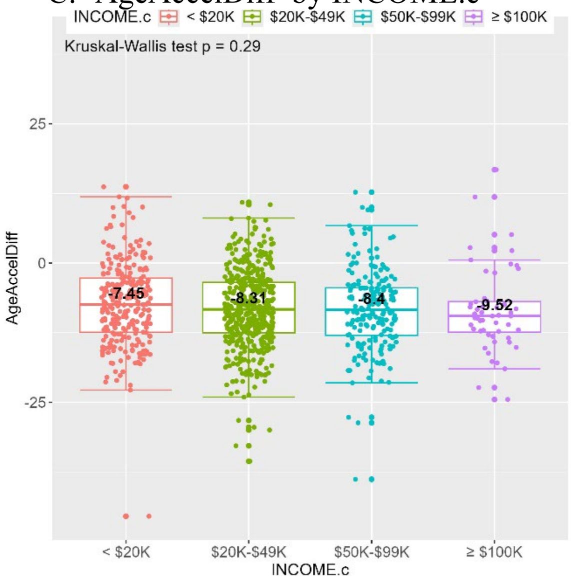

D. IEAA by INCOME.c

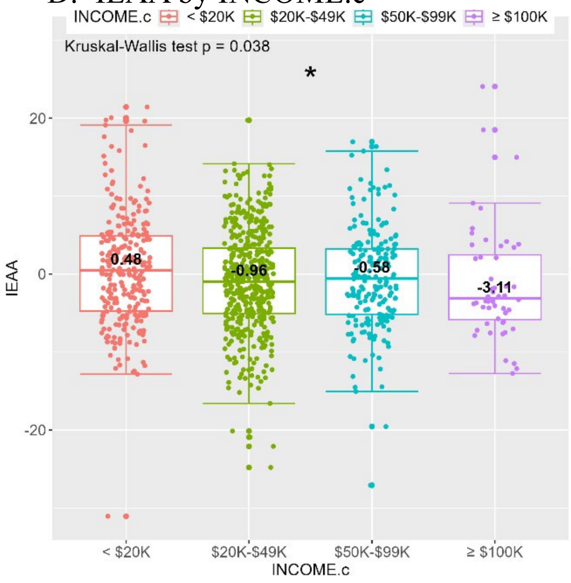

E. AgeAccelDiff by ANYINS

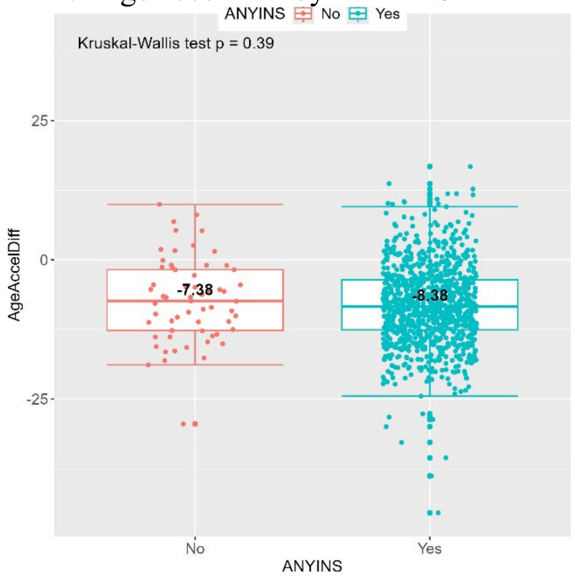

F. IEAA by ANYINS

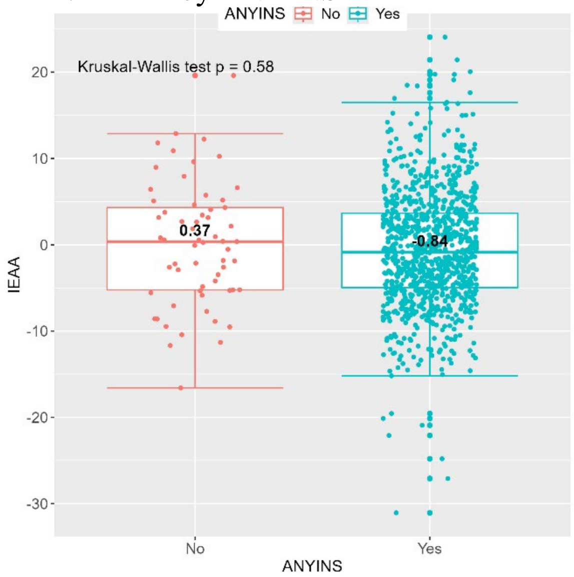

G. AgeAccelDiff by SOCSUPP

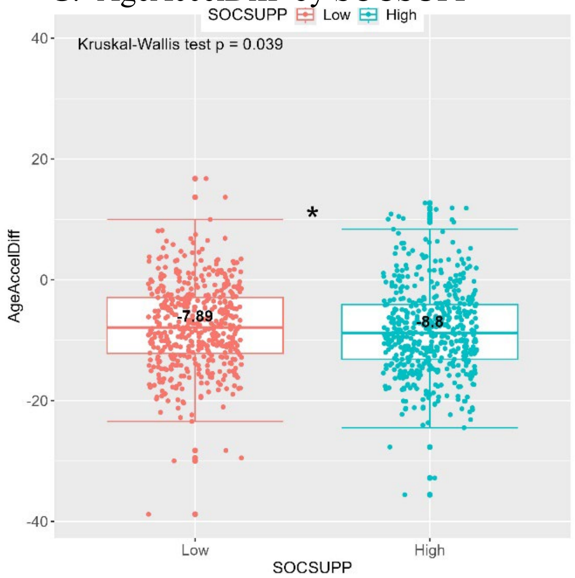

H. IEAA by SOCSUPP

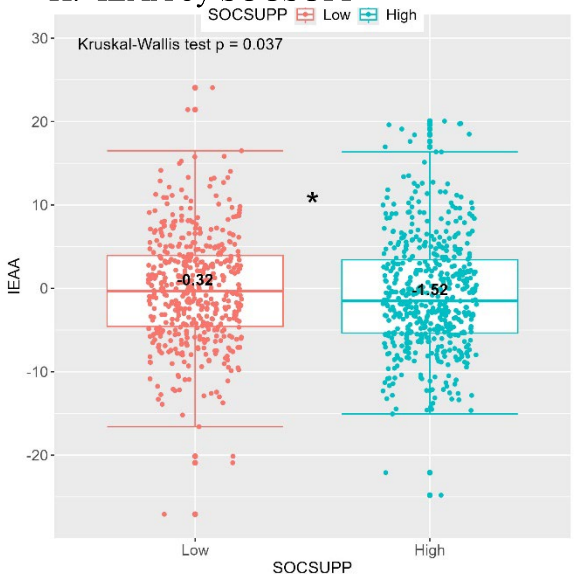

Figure S5. Analytic step 2, pathway 1 in HT.ch.y data: distribution of AgeAccelDiff and IEAA by social determinants of health. (AgeAccelDiff, epigenetic age acceleration as departure of DNAmAge from chronologic age; ANYINS, any insurance variable categorized; EDUC.c, education variable categorized; HT.ch.y, hypertension status change with two outcomes at annual visit 3 (improvement vs. no improvement); IEAA, intrinsic epigenetic age acceleration as residuals adjusted for cell composition; INCOME.c, family income variable categorized; SOCSUPP, social support construct categorized.)

A. AgeAccelDiff by EDUC.c

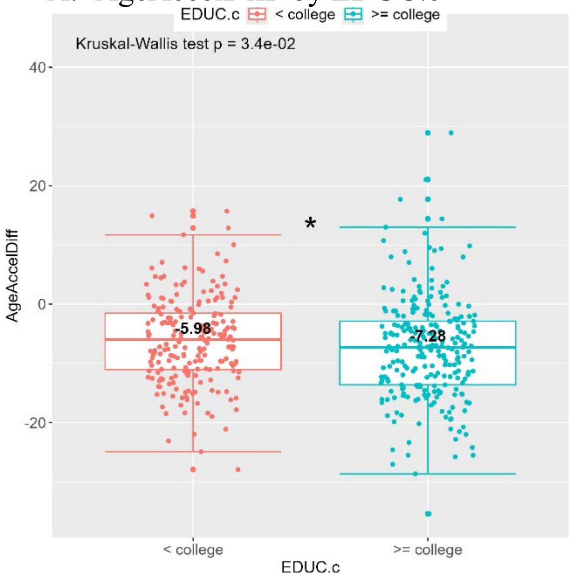

B. IEAA by EDUC.c

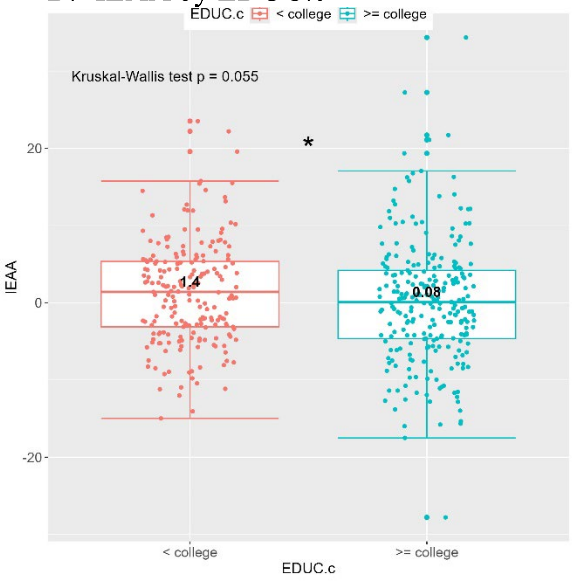

C. AgeAccelDiff by INCOME.c

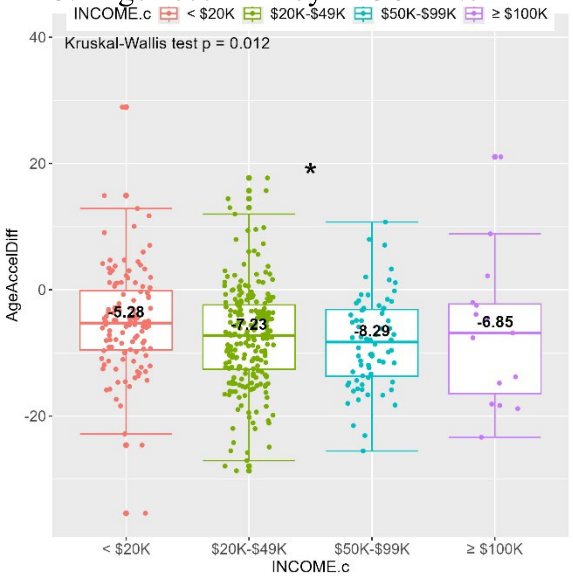

D. IEAA by INCOME.c

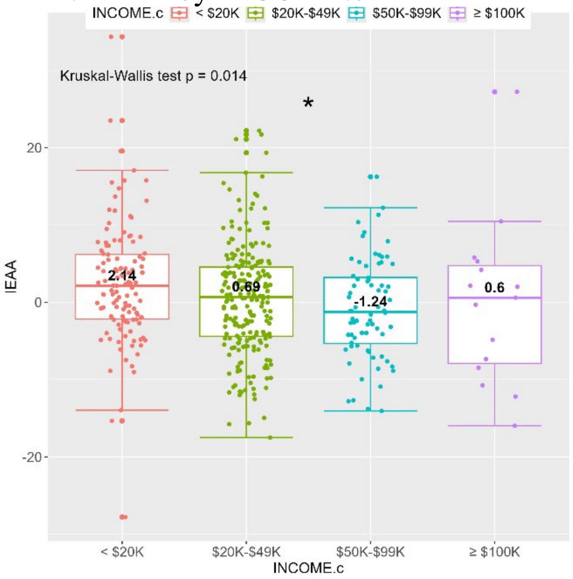

E. AgeAccelDiff by ANYINS

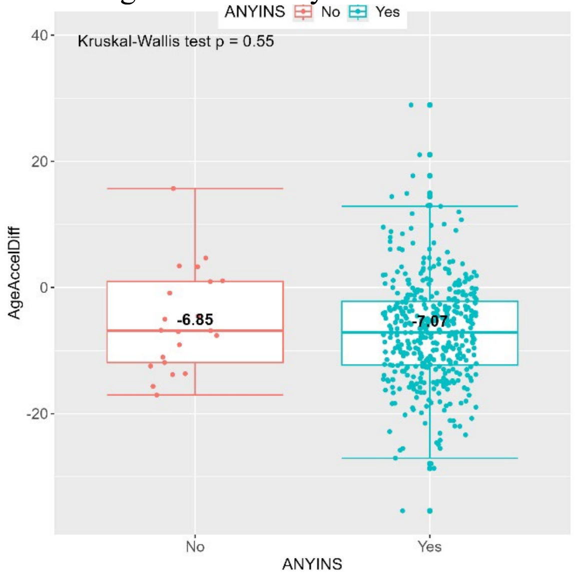

F. IEAA by ANYINS

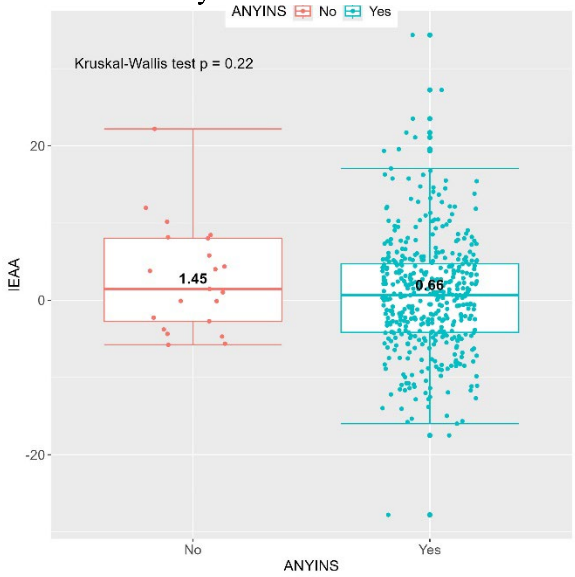

G. AgeAccelDiff by SOCSUPP

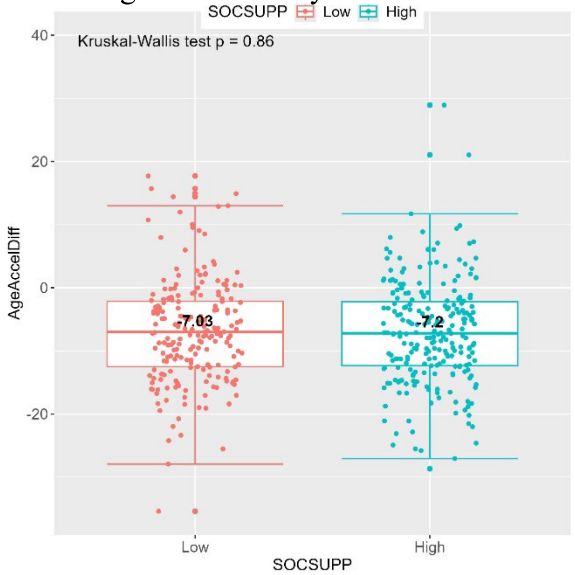

H. IEAA by SOCSUPP

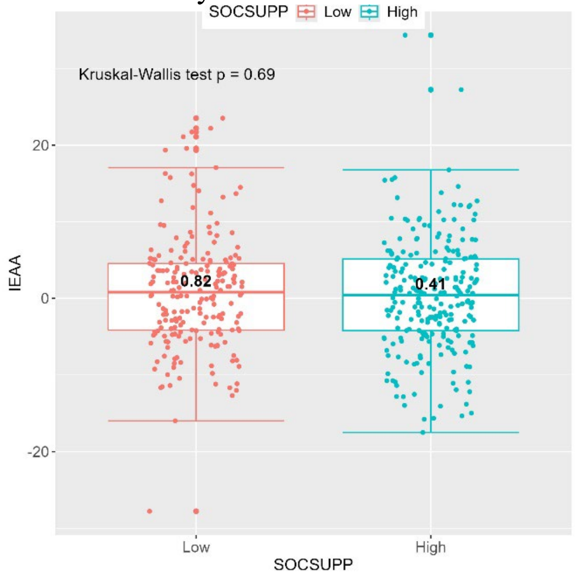

Figure S6. Analytic step 2, pathway 1 in HT.ch.all data: distribution of AgeAccelDiff and IEAA by social determinants of health. (AgeAccelDiff, epigenetic age acceleration as departure of DNAmAge from chronologic age; ANYINS, any insurance variable categorized; EDUC.c, education variable categorized; HT.ch.all, hypertension status change with all outcomes at annual visit 3 (no development or improvement vs. development or no improvement); IEAA, intrinsic epigenetic age acceleration as residuals adjusted for cell composition; INCOME.c, family income variable categorized; SOCSUPP, social support construct categorized.)

A. AgeAccelDiff by EDUC.c

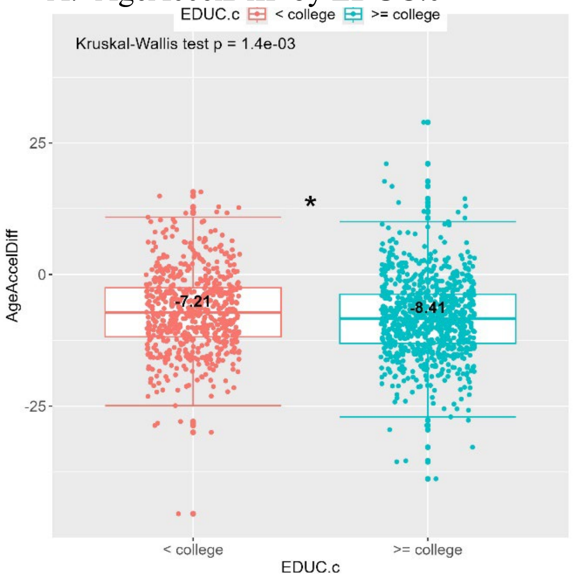

B. IEAA by EDUC.c

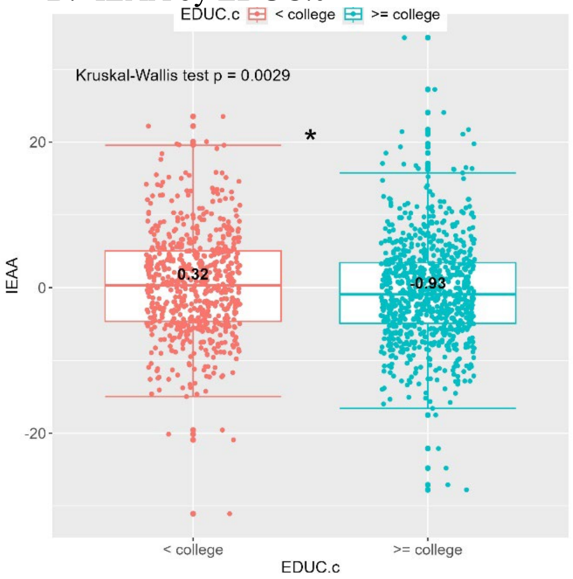

C. AgeAccelDiff by INCOME.c

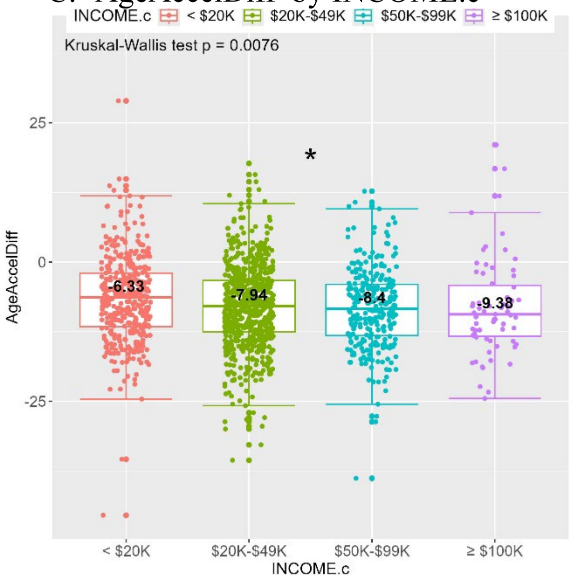

D. IEAA by INCOME.c

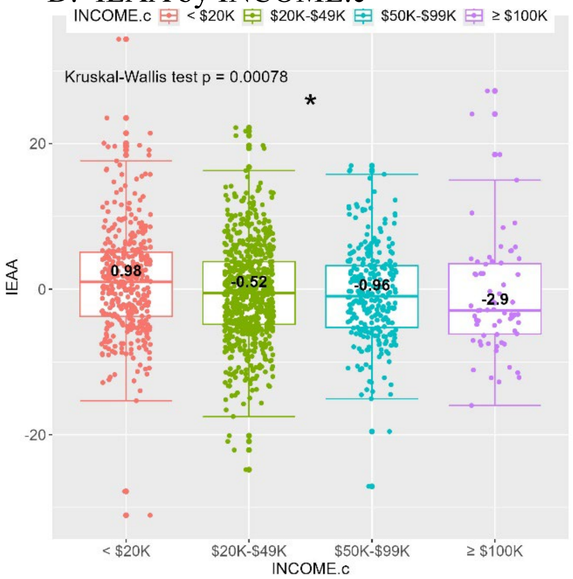

E. AgeAccelDiff by ANYINS

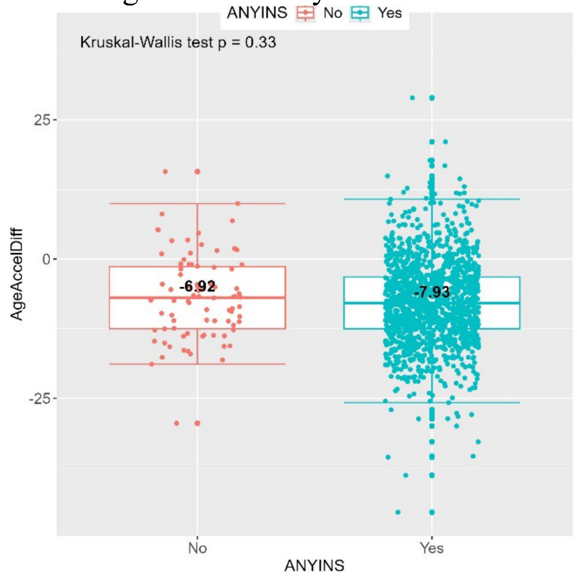

F. IEAA by ANYINS

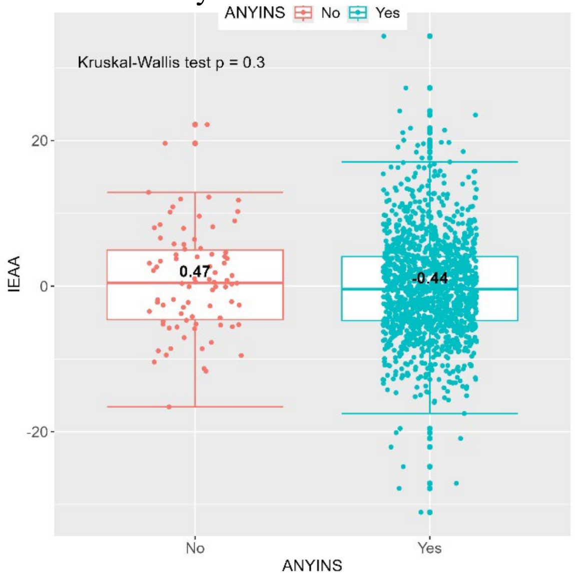

G. AgeAccelDiff by SOCSUPP

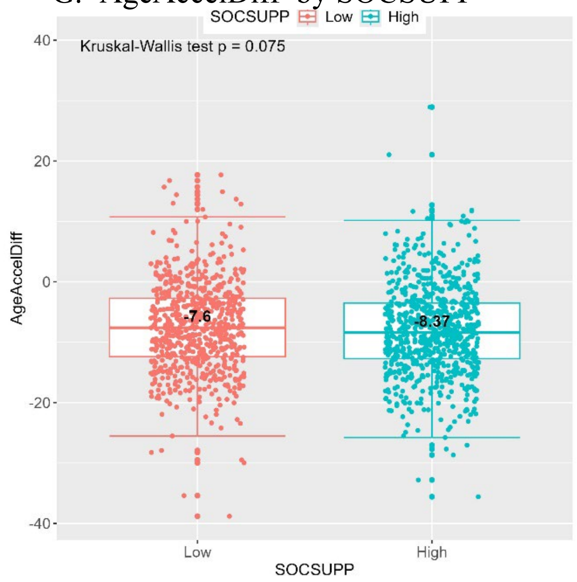

H. IEAA by SOCSUPP

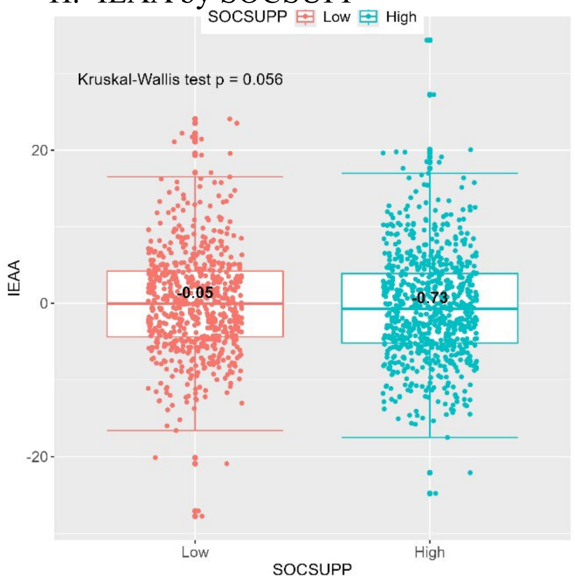

Figure S7. Analytic step 3: mediation effects of SDOH and biological aging marker in separate and joint analyses.(AgeAccelDiff, epigenetic age acceleration as departure of DNAmAge from chronologic age; DE, direct effect; HT.ch.all, hypertension status change with all outcomes at annual visit 3 (no development or improvement vs. development or no improvement); HYP, hypertension development; ID, indirect, mediation effect; IEAA, intrinsic epigenetic age acceleration as residuals adjusted for cell composition; SDOH, social determinant of health; TE, total effect.)

A. HYP outcome

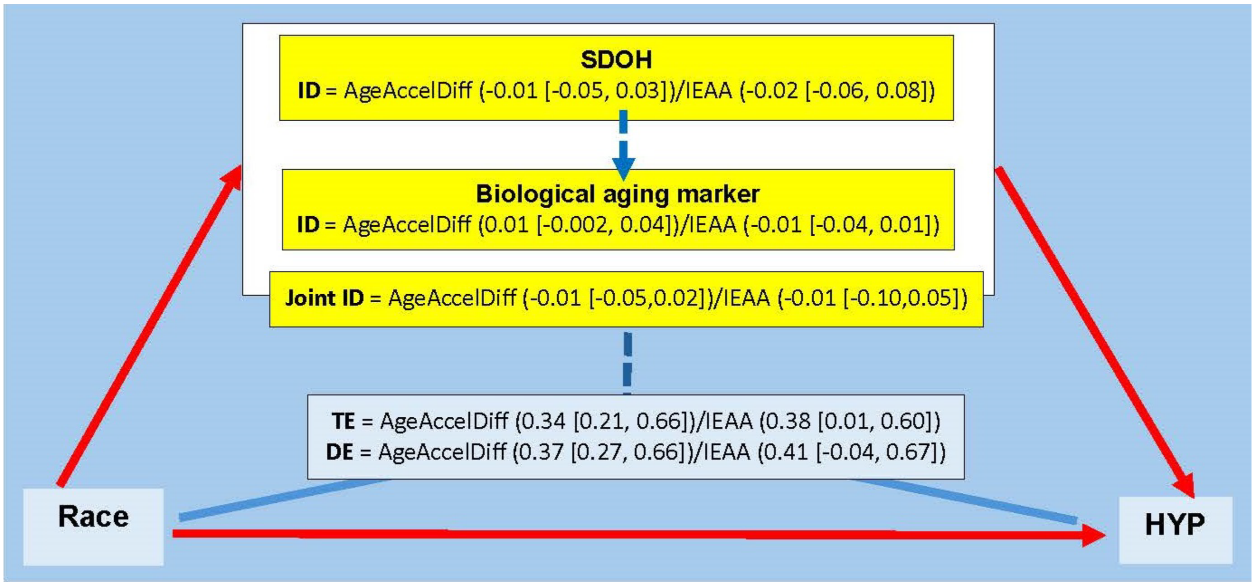

B. HT.ch.all outcome

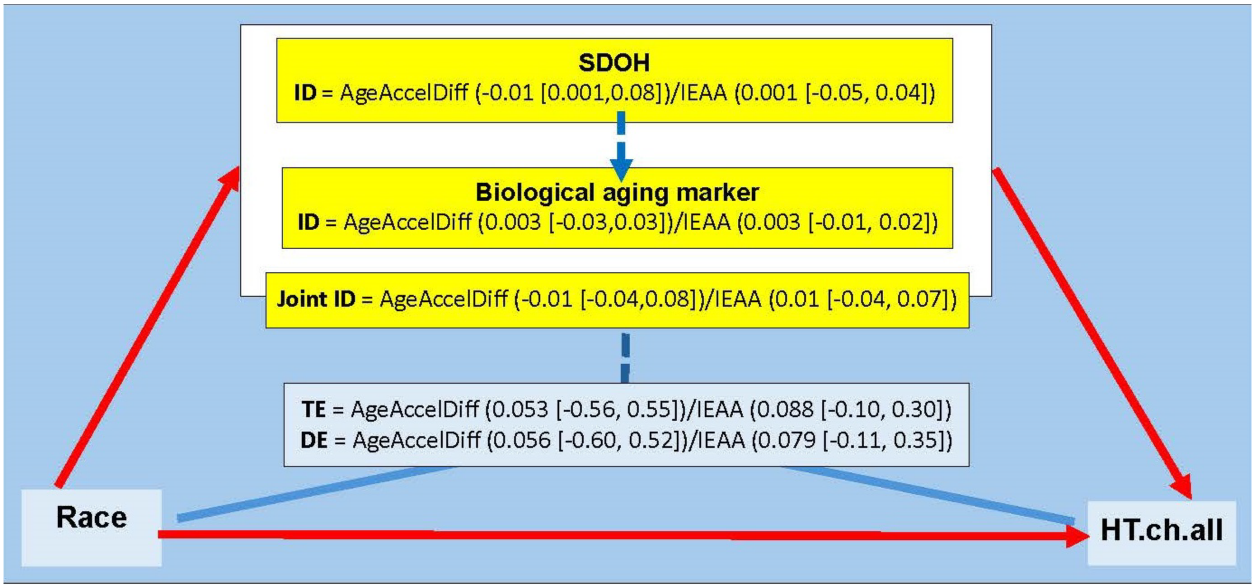

Supplement: Supplementary file 1 — Supplementary Material 1. [file 13148_2026_2117_MOESM1_ESM.pdf]
